# Socs36E Controls Niche Competition by Repressing MAPK Signaling in the *Drosophila* Testis

**DOI:** 10.1371/journal.pgen.1005815

**Published:** 2016-01-25

**Authors:** Marc Amoyel, Jason Anderson, Annabelle Suisse, Johanna Glasner, Erika A. Bach

**Affiliations:** 1 Department of Biochemistry and Molecular Pharmacology, New York University School of Medicine, New York, New York, United States of America; 2 The Helen L. and Martin S. Kimmel Center for Stem Cell Biology, New York University School of Medicine, New York, New York, United States of America; University of California, Los Angeles, UNITED STATES

## Abstract

The *Drosophila* testis is a well-established system for studying stem cell self-renewal and competition. In this tissue, the niche supports two stem cell populations, germ line stem cells (GSCs), which give rise to sperm, and somatic stem cells called cyst stem cells (CySCs), which support GSCs and their descendants. It has been established that CySCs compete with each other and with GSCs for niche access, and mutations have been identified that confer increased competitiveness to CySCs, resulting in the mutant stem cell and its descendants outcompeting wild type resident stem cells. *Socs36E*, which encodes a negative feedback inhibitor of the JAK/STAT pathway, was the first identified regulator of niche competition. The competitive behavior of *Socs36E* mutant CySCs was attributed to increased JAK/STAT signaling. Here we show that competitive behavior of *Socs36E* mutant CySCs is due in large part to unbridled Mitogen-Activated Protein Kinase (MAPK) signaling. In *Socs36E* mutant clones, MAPK activity is elevated. Furthermore, we find that clonal upregulation of MAPK in CySCs leads to their outcompetition of wild type CySCs and of GSCs, recapitulating the *Socs36E* mutant phenotype. Indeed, when MAPK activity is removed from *Socs36E* mutant clones, they lose their competitiveness but maintain self-renewal, presumably due to increased JAK/STAT signaling in these cells. Consistently, loss of JAK/STAT activity in *Socs36E* mutant clones severely impairs their self-renewal. Thus, our results enable the genetic separation of two essential processes that occur in stem cells. While some niche signals specify the intrinsic property of self-renewal, which is absolutely required in all stem cells for niche residence, additional signals control the ability of stem cells to compete with their neighbors. Socs36E is node through which these processes are linked, demonstrating that negative feedback inhibition integrates multiple aspects of stem cell behavior.

## Author Summary

Niches are specialized local environments that support stem cell self-renewal through the local production of short-range signals. In many tissues, resident stem cells compete with each other for niche access. Stem cells that receive multiple self-renewal cues have to integrate these discrete signals to prevent excessive competition or premature differentiation. Negative feedback inhibition between signaling pathways can provide a cohesive node through which such signals are interpreted. Here we show that a negative feedback inhibitor of the JAK/STAT pathway (Socs36E) acts as a brake on a second signaling pathway (MAPK). Stem cells lacking *Socs36E* have sustained MAPK signaling, which endows these cells with superior competitive skills that allow them to displace their normal neighbors and take over the entire niche. Our results show that dampening niche-derived signals is critical in maintaining balance in stem cell niches.

## Introduction

**Fig 1:**
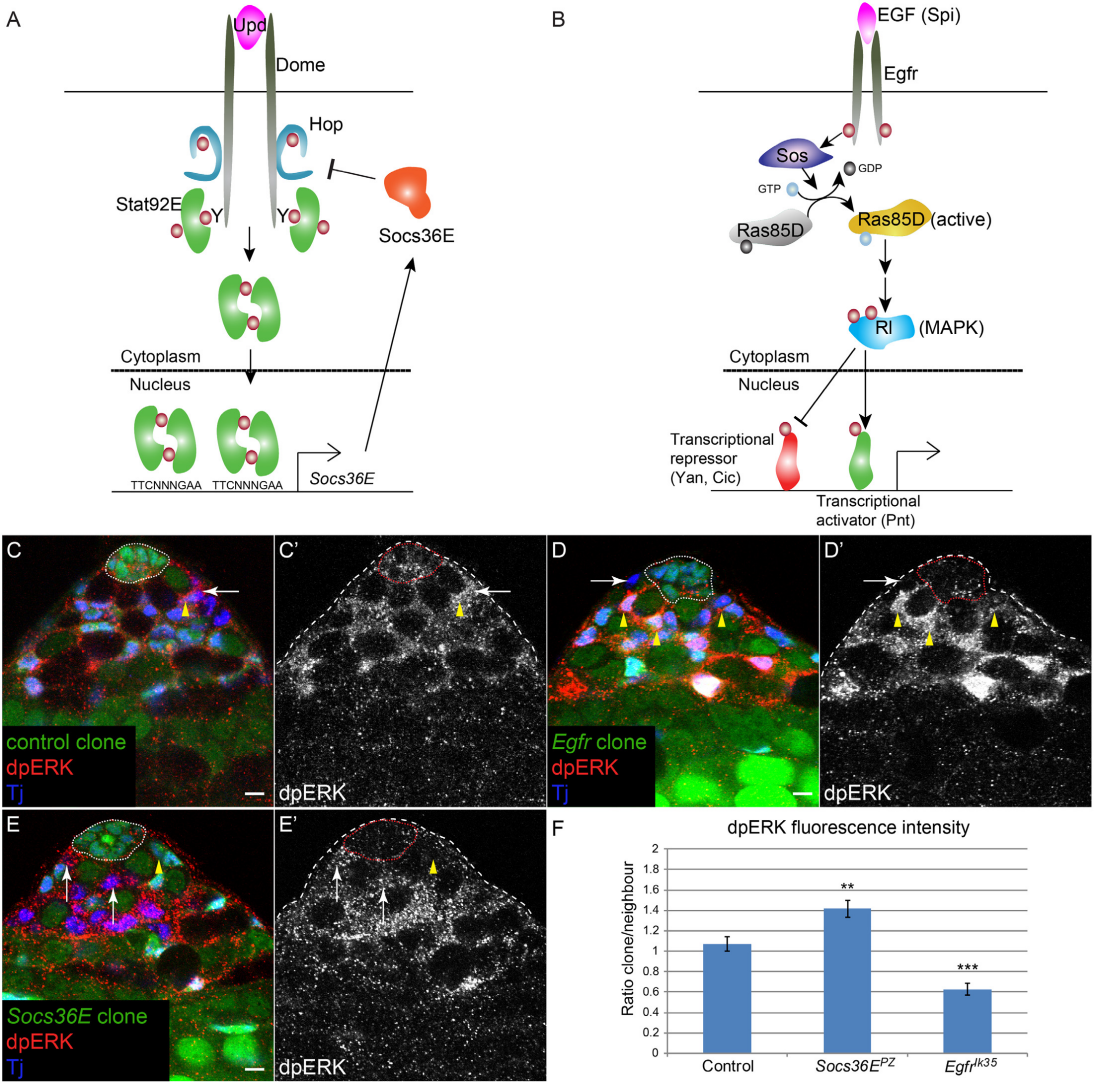
MAPK signaling is elevated in *Socs36E* mutant clones. A) Model of the *Drosophila* JAK/STAT pathway. The ligand Unpaired (Upd, magenta) is produced by hub cells and binds to and activates the receptor Domeless (Dome, gray) on the surface of CySCs. This results in activation of the JAK Hopscotch (Hop) (blue), leading to tyrosine phosphorylation (maroon circles) of Dome. The phosphorylated cytoplasmic domain of the receptor serves as a docking site for a Stat92E dimer (green). Stat92E is phosphorylated leading to the generation of an active Stat92E dimer that translocates to the nucleus, binds to a consensus TTCNNNGAA site, and alters gene expression. *Socs36E* is one of the best-characterized Stat92E target genes and encodes a negative regulator of JAK/Receptor activity (orange). B) Model of the MAPK pathway. The EGF ligand Spitz (Spi, magenta) is produced by germ line cells. Spi activates the EGF receptor (Egfr, dark gray) on the surface of CySCs, which triggers the canonical MAPK pathway. Activation of Egfr leads to the recruitment of Son of Sevenless (Sos, dark blue), a guanine exchange factor for Ras85D (Ras) that converts Ras from an inactive GDP-loaded form (light gray) to an active GTP-loaded form (yellow). Ras activates Rolled (Rl, blue), a *Drosophila* MAPK, which inhibits transcriptional repressors (orange) Yan and Capicua (Cic) and activates Pointed (Pnt), a transcriptional activator (green). C-E) MAPK activity (dpERK, red) in control clones, *Egfr* mutant clones and *Socs36E* mutant clones. Clones are negatively-marked and are identified by the lack of GFP. C) dpERK can be detected in a control CySC clone (C,C’, arrow), which lacks GFP (green), at similar levels to a neighboring wild type CySC (C,C’, arrowhead). These data indicate that MAPK activity is normally present in CySCs. MAPK activity (dpERK) is decreased in an *Egfr* mutant CySC (D,D’, arrow), compared to neighboring wild type CySCs (D,D’, arrowheads). E) dpERK is elevated in *Socs36E* mutant CySCs (E,E’ arrows) compared to a neighboring wild type CySC (E,E’, arrowhead). F) Quantification of fluorescence intensity in clones, expressed as a ratio of intensity within a CySC compared to that of its immediate unmarked wild type neighbor CySC. Asterisks indicate statistical significance ** P<0.01 and *** P<0.001. Tj is blue in C-E. The hub is outlined by a dotted line. Scale bar = 5 μM.

**Fig 2:**
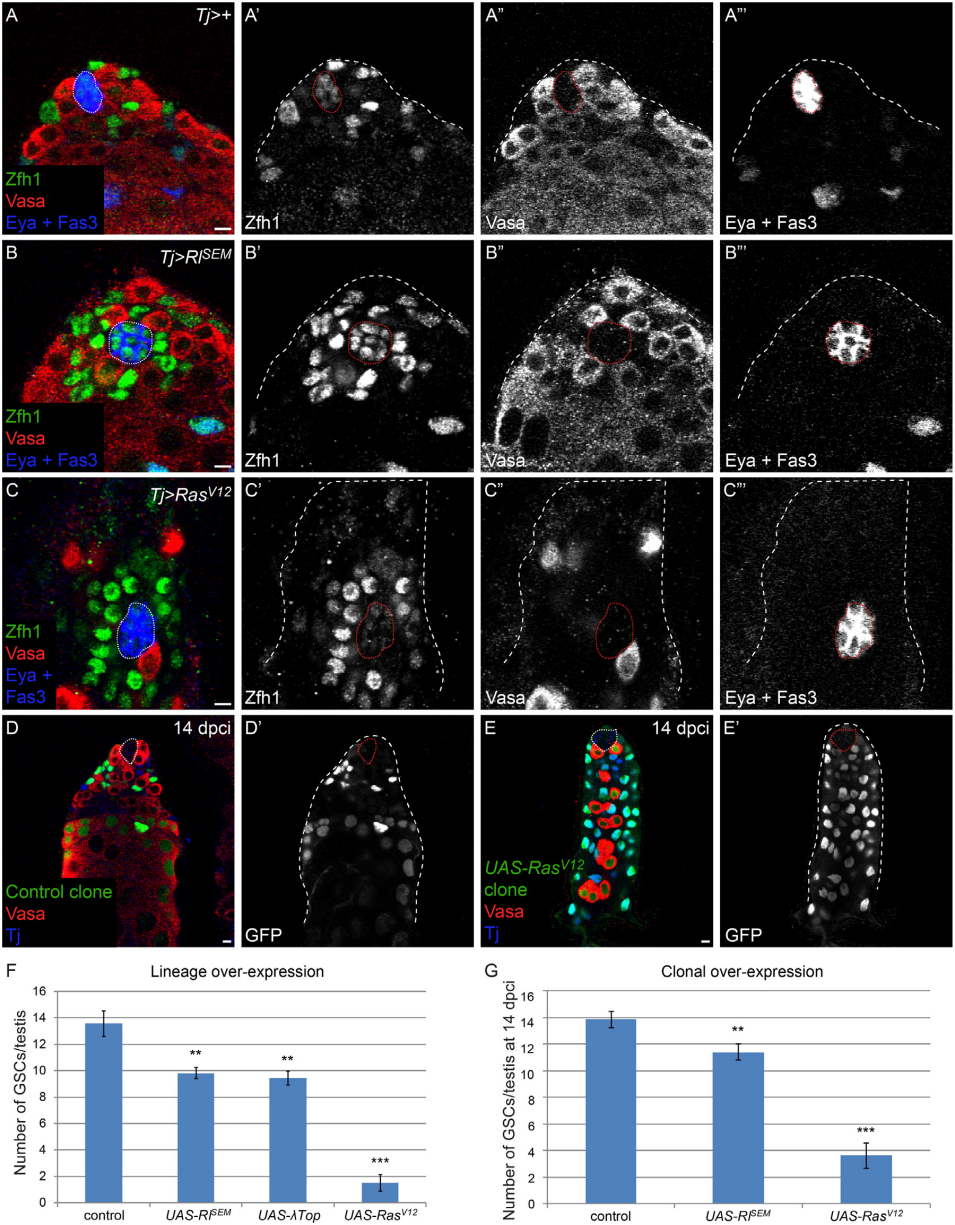
MAPK gain-of-function in CySCs causes them to out-compete GSCs at the niche. A) A normal complement of Vasa-positive GSCs and Zfh1-positive CySCs at the niche and Eya-positive differentiating cyst cells is observed in a control a *Tj-Gal4* (*Tj>+*) testis. B) GSCs are displaced by CySCs over-expressing dominant-active form of MAPK (Rl^SEM^). C) GSCs are almost entirely out-competed by CySCs over-expressing a dominant-active Ras (Ras^V12^). In this image, only one GSC remains in contact with the niche and there are numerous CySCs immediately contacting hub cells. We note that the position of the hub frequently shifts slightly basally in *Tj>Ras*^*V12*^ over-expressing testes. D) A testis with control MARCM clones at 14 dpci, identified by the expression of GFP within the clone. Note that some CySCs and their differentiating progeny are clonally marked with GFP and there is a normal complement of GSCs at the niche. E) A testis with *UAS-Ras*^*V12*^ MARCM clones at 14 dpci. The entire somatic lineage is clonally marked, indicating that Ras^V12^-expressing CySCs out-competed wild type CySCs. There are two GSCs in contact with the niche, indicating that Ras^V12^-expressing CySCs also out-competed most GSCs in this testis. Germ line differentiation is also perturbed, presumably because somatic cells with high Ras activity cannot adequately support gonial cells [44]. F) Graph displaying the number of GSCs in control (*Tj-Gal4*), *Tj>Rl*^*SEM*^, *Tj>λTop*, and *Tj>Ras*^*V12*^ testes. GSC number is moderately but significantly reduced in *Tj>Rl*^*SEM*^ and *Tj>λTop* and substantially reduced in *Tj>Ras*^*V12*^ testes. G) Graph displaying the number of GSCs in testes containing control, Rl^SEM^-expressing and Ras^V12^ -expressing CySC clones. Note the significant drop in GSCs when CySC clones expressing *UAS-Rl*^*SEM*^ and *UAS-Ras*^*V12*^ are present. **P< 0.01, ***P<0.001. Vasa is red in all panels. Zfh1 is green and Fas3 and Eya are blue in A-C. MARCM clones, which express GFP, are green and Tj is blue in D and E. The hub is outlined by a dotted line. Scale bar = 5 μM.

Stem cell niches are complex environments that provide support for stem cells through molecular signals. Several well-characterized niches provide not just one but multiple signals which stem cells must integrate and interpret in order to remain at the niche and self-renew [1]. How this integration is achieved is not well understood at present. Furthermore, in order to maintain the appropriate number of stem cells and the homeostatic balance between self-renewal and differentiation, it is necessary that self-renewal cues be present in limiting amounts or that their activity be dampened to prevent excessive accumulation of stem cells. One general feature of many signal transduction pathways is the presence of feedback inhibitors [2–4]. These are dampeners of signaling, transcriptionally induced by the signaling itself, that prevent signal levels from being aberrantly high. One such family of feedback inhibitors is the Suppressor of Cytokine Signaling (SOCS) proteins, which were identified as inhibitors of JAK/STAT (Janus Kinase/Signal Transduction and Activator of Transcription) signal transduction, and are SH2- and E3-ligase domain-containing proteins (Fig 1A and [2]). The SH2 domain binds phosphorylated (i.e., activated) signal transduction components and the E3-ligase targets them for degradation by Ubiquitin-dependent proteolysis. In mammals, SOCS proteins can thus inhibit several tyrosine kinase-dependent signaling pathways, including JAK/STAT and Mitogen-Activated Protein Kinase (MAPK) [2,5].

The *Drosophila* testis is an ideal model system to study questions of signal regulation and integration in stem cells [6]. The testis niche, called the hub, supports two stem cell populations. The first, germ line stem cells (GSCs), gives rise to sperm after several transit-amplifying divisions leading up to meiosis. The second, somatic cyst stem cells (CySCs), gives rise to cyst cells, the essential support cells for germ line development. Many ligands for signaling pathways are produced by the hub, including the JAK/STAT pathway agonist, Unpaired (Upd), the Hedgehog (Hh) pathway ligand Hh and the Bone Morphogenetic Protein (BMP) homologs Decapentaplegic (Dpp) and Glass Bottom Boat (Gbb) [7–11]. The latter two signals are also produced by CySCs and are required in GSCs for self-renewal, indicating that CySCs constitute part of the niche for GSCs along with the hub [10,12]. CySCs require JAK/STAT and Hh activity for self-renewal [8,13–15].

CySCs and GSCs compete for space at the niche, a phenomenon that was revealed by the analysis of testes lacking the JAK/STAT feedback inhibitor *Socs36E* [16,17]. In these animals, excessive JAK/STAT activity was detected in CySCs, and *Socs36E* mutant CySCs displaced the resident wild type GSCs. Additionally, we recently showed that CySCs with sustained Hh signaling or sustained Yorkie (Yki) activity also outcompeted neighboring wild type GSCs, indicating that several signaling pathways can control niche competition [18]. Moreover, we showed that prior to out-competing GSCs, mutant CySCs displaced neighboring wild type CySCs, indicating that both intra- (CySC-CySC) and inter-lineage (CySC-GSC) competition take place in the testis. While the two types of competition appear related, in that one precedes the other, there are instances in which only intra-lineage competition takes place [19]. While the competitive phenotype of *Socs36E* mutant CySCs was ascribed to increased JAK/STAT signaling [16,17], we were surprised to find that clonal gain-of-function in JAK/STAT signaling in CySCs did not induce competitive behavior, and we concluded that loss of *Socs36E* did not mimic increased JAK/STAT signaling in CySCs [18].

Here, we address whether other mechanisms could account for the competitive behavior of *Socs36E* mutant CySCs. Because SOCS proteins can inhibit MAPK signaling in cultured cells and in *Drosophila* epithelial tissues [5,20,21], we examined if Socs36E repression of MAPK signaling underlied the *Socs36E* competitive phenotype. Indeed, we find that Socs36E inhibits MAPK signaling in CySCs during self-renewal, and that gain of MAPK activity induces CySCs to outcompete wild type CySCs and GSCs at the niche. We dissect the genetic relationship between Socs36E and the MAPK and JAK/STAT pathways and show that loss of *Socs36E* can compensate for decreased self-renewal signaling within CySCs. Thus, we show that CySCs integrate multiple self-renewal signals through the use of a feedback inhibitor that controls at least two signaling pathways regulating stem cell maintenance at the niche.

## Results

### Gain-of-function in MAPK resembles loss of *Socs36E*

Loss of *Socs36E* in the somatic lineage leads to the displacement of GSCs at the niche by mutant CySCs [16]. Although Socs36E is a well-described inhibitor of JAK/STAT signaling [22–25], we previously determined that elevating JAK/STAT pathway activity in CySCs did not cause the displacement of GSCs [18]. Therefore, we asked whether another signaling pathway known to be inhibited by Socs36E—the MAPK pathway—could be responsible for the niche colonization phenotype by *Socs36E* mutant CySCs in the testis. Previous work has found that over-expression of Socs36E inhibited MAPK activity and conversely Socs36E knock down enhanced MAPK-dependent tumorigenesis [20–22,24]. Increased MAPK activity in Socs36E-depleted cultured cells suggested that Socs36E directly regulated the MAPK pathway [20], but whether this occurs in vivo is yet to be established. MAPK signaling is activated by several extracellular ligands, the best characterized of which are epidermal growth factors (EGFs), acting through the EGF receptor (Egfr) (reviewed in [3]). Upon ligand binding to Egfr, intracellular adaptors recruit the Ras guanine exchange factor (GEF) Son of Sevenless (Sos). Sos activates Ras and initiates a phosphorylation cascade resulting in MAPK (called Rolled (Rl) in *Drosophila*) activation and subsequent gene transcription alterations through several ETS domain-containing transcription factors (Fig 1B). EGF ligands are present in the testis; germ cells produce Spitz (Spi) while somatic cells express *vein* [26,27].

First, we tested whether MAPK signaling levels were regulated by Socs36E in the testis. We induced mutant clones for *Socs36E* and stained for phosphorylated MAPK (dpERK), an established readout for pathway activity [28]. In testes with control clones, we observed dpERK staining in CySCs as well as in differentiating cyst cells (Fig 1C, arrow for stem cell clone and arrowhead for unmarked CySC). The dpERK staining in CySCs is dependent on Egfr activity because the dpERK signal was autonomously lost in *Egfr* mutant clones (Fig 1D, compare arrow to arrowheads). By contrast, we found that dpERK staining was autonomously increased in *Socs36E* mutant CySCs (Fig 1E, compare arrows to arrowhead). Because dpERK staining was variable, we compared the fluorescence intensity of the marked clone with that of its immediate wild type CySC neighbor (Fig 1F). This analysis revealed a significant decrease in dpERK intensity in *Egfr* mutant clones (P = 0.0008) and a significant increase in *Socs36E* mutant clones (P = 0.0071). While a previous study reported that *Socs36E* did not regulate dpERK using *Socs36E* mutant testes [17], our clonal analysis provides better resolution and allows for direct comparison of mutant and wild type CySCs in the same tissue. Taken together, these data suggest that Socs36E negatively regulates the MAPK pathway in normal CySC function, in addition to its known role in repressing JAK/STAT activity.

Next, we sought to establish whether increased MAPK signaling in somatic cells in the testis could cause CySCs to outcompete GSCs for space at the niche and recapitulate the loss of GSCs observed in *Socs36E* mutant testes (S1 Table and [16]). We used the somatic cell driver *Traffic jam* (*Tj*)-*Gal4* to hyper-activate MAPK in all CySCs and their lineage. In controls, we found 13.6 GSCs contacting the hub, and the nuclei of CySCs, marked with Zfh1, were visible behind the GSCs (Fig 2A and 2F). When we over-expressed either a dominant-active form of the EGF receptor (λTop) or of MAPK, Rolled (Rl^SEM^), we observed CySCs contacting the hub directly in the place of GSCs (Fig 2B and 2F). We counted the number of GSCs in these genotypes and found that hyper-activation of MAPK in CySCs resulted in a significant loss of GSCs non-autonomously (Fig 2F, S1 Table, 13.6 in control vs 9.4 in *UAS-λTop* and vs 9.8 in *UAS-Rl*^*SEM*^, P<0.0013 and P<0.0025, respectively). We note that we did not see an increase in βPS-integrin when MAPK signaling was hyper-activated in CySCs or when *Socs36E* was lost from these cells (S1 Fig). Additionally, when we over-expressed a very strong activator of the pathway, Ras^V12^, using *Tj-Gal4* we observed a dramatic loss of GSCs (1.5 GSCs in *UAS-Ras*^*V12*^ testes) (Fig 2C and 2F, S1 Table), indicating that the strength of competition between CySCs and GSCs depends on the level of MAPK activation in CySCs.

Finally, as these experiments tested CySC-GSC competition using lineage-wide over-expression, we wanted to determine whether a single CySC clone with increased MAPK activation could outcompete wild type CySCs (CySC-CySC competition) and GSCs (CySC-GSC competition) for space at the niche. We used the MARCM technique [29] to generate control clones, or clones that over-expressed either Rl^SEM^ or Ras^V12^. At 14 days post clone induction (dpci), we observed that wild type clones labelled a variable fraction of CySCs, consistent with CySCs undergoing stochastic loss and replacement (Fig 2D and [18]). However, clones in which MAPK was hyper-activated had replaced most wild type CySCs by 14 dpci (Fig 2E) and also had outcompeted resident GSCs, leading to a significant reduction in GSC numbers (Fig 2G). These phenotypes closely resemble the effects that we and others observed in *Socs36E* mutant clones (see below and [16]). Specifically, we observed 13.9 GSCs in testes with control clones and 8.4 GSCs in testes with *Socs36E* clones (P<5.2x10^-8^). In contrast, gain-of-function of JAK/STAT signaling in CySC clones did not lead to GSC loss [18]. Together, our results suggest that both CySC-CySC and CySC-GSC competition induced by *Socs36E* loss is due primarily to the increase in MAPK activity in these cells, rather than that of JAK/STAT.

### MAPK pathway activity regulates CySC numbers

The results presented above suggest that CySCs undergo MAPK signaling and are responsive to changes in levels of its activity. Indeed, labelling with dpERK antibody reveals that the MAPK signaling pathway is active in wild type CySCs (Fig 1C and [26,27]). Although MAPK has been shown to be required during cyst cell differentiation in the testis [26,27,30,31], its role in the CySCs themselves is unclear. Previous work has reported that persistent *Egfr* or *raf* mutant clones are not recovered, but there is a debate as to whether this reflects a requirement for MAPK signaling in CySCs during self-renewal [17,27,30].

**Fig 3:**
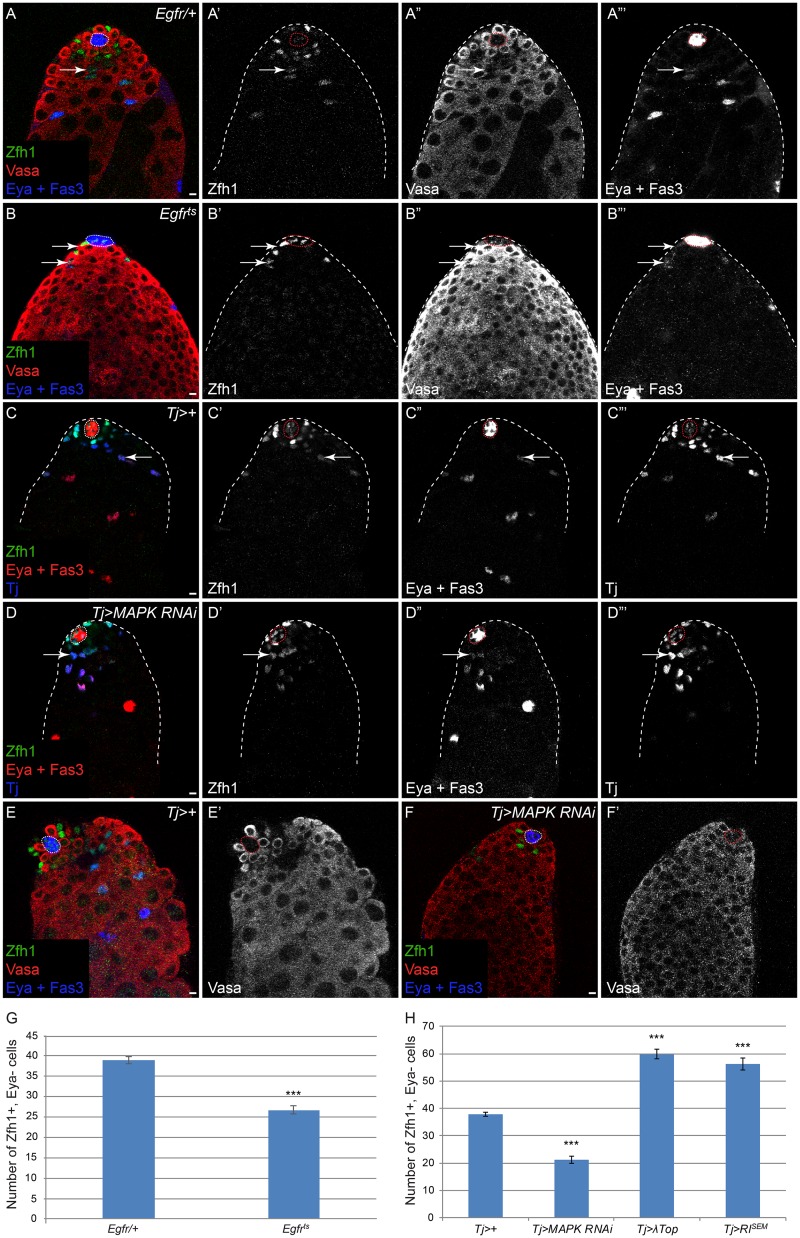
MAPK signaling regulates CySC numbers. A) A representative testis from an *Egfr*/+ animal contains a rosette of GSCs in contact with the niche surrounded by a row of Zfh1-positive CySCs. The arrow highlights a differentiating cyst cell that has started to express Eya. B) A testis from an *Egfr*^*ts*^ animal that was upshifted to the restrictive temperature of 29°C for 10 days. Note the accumulation of many small Vasa-positive early germ cells and a reduction in the number of Zfh1-positive cells. Arrows mark somatic cells close to the niche that have turned on expression of the differentiation marker Eya. C) Markers of the somatic lineage in a control *Tj-Gal4* (*Tj>+*) testis. Tj (blue) is expressed at low levels in the hub and at higher levels in CySCs and early cyst cells. Zfh1-positive CySCs (green) are observed near the niche and Eya (red) begins to be expressed in differentiating cyst cells. Arrow marks a differentiating cyst cell that is several rows away from the niche and that has upregulated expression of Eya. D) *Tj>MAPK RNAi* testis. Note the reduction in Zfh1-positive cells and upregulation of Eya in somatic cells close to the niche, which is not observed in controls (compare to arrow in C). E) A normal complement of Vasa-positive GSCs and large differentiating spermatogonia in a control *Tj-Gal4* (*Tj>+*) testis. F) In a *Tj>MAPK RNAi* testis, there is a block in germ cell differentiation, an accumulation of many small early germ cells and a total lack of differentiating spermatogonia, similar to *Egfr*^*ts*^ testes at the restrictive temperature (see B). G,H) Graphs showing the number of CySCs, defined as Zfh1-positive, Eya-negative cells, in *Egfr*/+, *Egfr*^*ts*^, control (*Tj-Gal4*), *Tj>MAPK RNAi*, *Tj>λTop*, and *Tj>Rl*^*SEM*^ testes. (G) Note a significant drop in CySCs in *Egfr*^*ts*^ testes compared to *Egfr/+*. H) Note significant decrease in CySCs in *Tj>MAPK RNAi* testes compared to control and a significant increase in the number of CySCs in *Tj>λTop* and *Tj>Rl*^*SEM*^ testes (see S1 Table for “n” values). Vasa is red in A,B,E,F. Zfh1 is green in all panels. Fas3 and Eya are blue in A,B,E,F and red in C,D. The hub is outlined by a dotted line. Scale bar = 5 μM.

In order to clarify the role of MAPK in CySCs, we addressed whether MAPK signaling affected CySC numbers and self-renewal. First, we examined testes from flies carrying a temperature-sensitive mutation in *Egfr* in *trans* to a loss-of-function allele (referred to as *Egfr*^*ts*^). When shifted to the restrictive temperature, these testes displayed the previously-described phenotype of a block in germ cell development, resulting in many small germ cells throughout the testes and a complete lack of differentiated spermatid fibers (Fig 3A and 3B and [27,30]). Notably, these testes also have fewer somatic cells near the hub (Fig 3A and 3B). We labelled these somatic cells with Zfh1 to mark the CySCs and their offspring and with Eya to mark differentiated somatic cells. In *Egfr*^*ts*^ testes, Eya expression was observed in somatic cells adjacent to the hub, suggesting that CySCs differentiate early in the absence of MAPK signaling (Fig 3B, arrows), corroborating prior observations [30,32]. We counted the number of CySCs (defined as Zfh1-positive, Eya-negative cells) in these samples and found that there were significantly fewer CySCs in *Egfr*^*ts*^ testes compared to control (Fig 3G, S1 Table, P<2.7x10^-12^). It was important to exclude Eya-expressing cells from the Zfh1 pool in this analysis because a prior study using only one somatic marker did not note a difference in somatic cell number when MAPK was decreased [32].

To determine whether the requirement for MAPK in maintenance of CySCs is autonomous to the somatic lineage, we used *Tj-Gal4* to over-express an RNAi against MAPK (Fig 3D). These testes displayed a phenotype similar to that which we observed in testes in which the entire animal was mutant for *Egfr* (Fig 3B). In *Tj>MAPK RNAi* testes, Eya-expressing cells were present close to the hub (Fig 3D”, arrow). However, in control *Tj>+* testes, Eya-expressing cells were located several cell diameters from the hub (Fig 3C”, arrow). We counted significantly fewer Zfh1-positive, Eya-negative CySCs when MAPK signaling was inhibited autonomously within the somatic lineage (Fig 3H, S1 Table, P<2.3x10^-12^). Moreover, as expected, proper germ cell development was inhibited in *Tj>MAPK RNAi* testes, resulting in an accumulation of small early germ cells (Fig 3F). Conversely, when we hyper-activated MAPK in somatic cells by over-expressing λTop or Rl^SEM^, there were significantly more Zfh1-expressing, Eya-negative cells (Fig 3H, S1 Table, P<1.4x10^-11^ for *UAS-λTop* and P<1.0x10^-7^ for *UAS-Rl*^*SEM*^). Taken together, these experiments indicate that MAPK acts autonomously within the somatic lineage to regulate CySC numbers.

### MAPK signaling is required autonomously for CySC self-renewal

**Fig 4:**
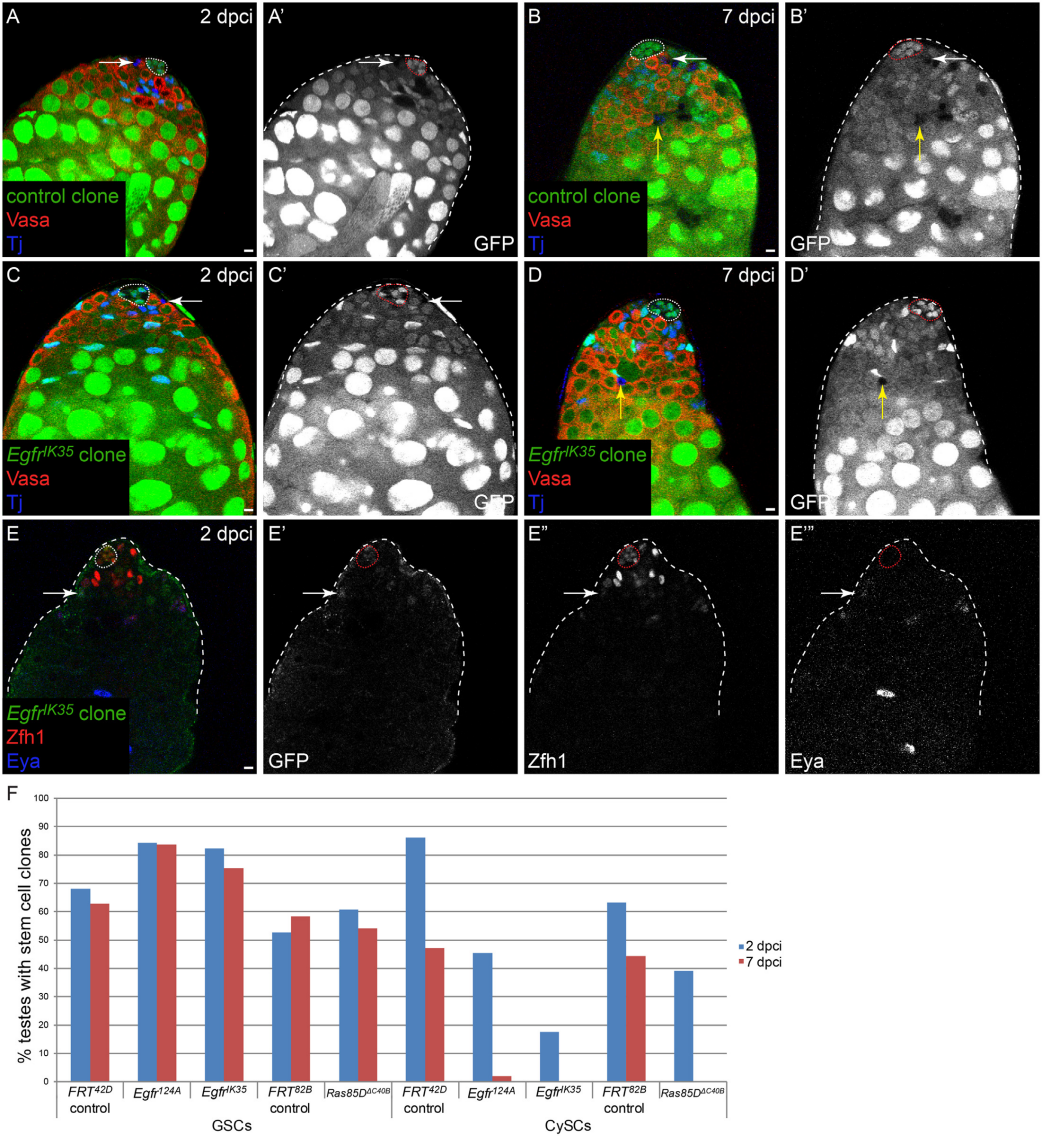
MAPK is required autonomously for CySC self-renewal. In A-D, clones are marked by the absence of GFP and in E, MARCM clones are marked by the presence of GFP. A) Testis with a control CySC clone (arrow) at 2 dpci. B) Testis with a control CySC clone at 7 dpci (B,B’, white arrow). Yellow arrow denotes a labeled differentiating cyst cell. C) Testis with an *Egfr* mutant clone (arrow) at 2 dpci, indicating that clones of this genotype can be induced. D) At 7 dpci, *Egfr* mutant CySC clones cannot be recovered. However, the differentiating offspring of these mutant clones are observed (yellow arrow). E) At 2 dpci, an *Egfr* mutant CySC (arrow) has already begun to differentiate as it has low levels of Zfh1 and has started to express Eya. F) Graph showing clone recovery rates for negatively-marked clones (mutant GSCs and mutant CySCs) at 2 and 7 dpci. See Table 1 for “n” values. Loss of *Egfr* or *Ras* does not affect the maintenance of GSCs. By contrast, CySC clones lacking either gene have reduced clone recovery rates at 2 dpci and are not maintained at 7 dpci. Vasa is red and Tj is blue in A-D. Zfh1 is red and Eya is blue in E. The hub is outlined by a dotted line. Scale bar = 5 μM.

As not all CySCs were lost when we inhibited MAPK within the whole somatic lineage, we generated mutant clones for components of the MAPK pathway to determine whether CySC clones with compromised MAPK signaling were able to self-renew. We generated both positively-marked and negatively-marked clones of several alleles of *Egfr* and the *Drosophila* Ras, *Ras85D*, and scored the presence of marked stem cells at 2 dpci, to verify that mutant clones could be induced, and at 7 dpci, to assess the ability of the mutant clones to self-renew at the niche. *Egfr* or *Ras85D* mutant GSCs were recovered and maintained at similar rates to control GSCs (Fig 4F, Table 1 for negatively-marked clones, S2 Table for positively-marked clones). However, CySCs mutant for *Egfr* or *Ras85D* were recovered less well at 2 dpci and were not maintained by 7 dpci (Fig 4F, Tables 1 and S2). We note that another group reported that *Ras85D*^Δ*C40B*^ null mutant clones were recovered at higher rates than control clones [17]. However, our results that CySCs lacking *Ras85D* function do not self-renew are supported by our use of multiple alleles of several pathway components (see below). Moreover, our results are consistent with prior reports that persistent *Egfr* or *raf* mutant somatic clones were not recovered [27,30]. We determined the fate of the clones that were induced but not recovered at later time points. We were able to detect *Egfr* mutant clones that expressed the differentiation marker Eya by 2 dpci (Fig 4E, arrow marks a positively-labeled clone), suggesting that *Egfr* mutant clones differentiate rapidly. Thus, our results indicate that MAPK activity is required autonomously in CySCs for self-renewal and that MAPK-deficient stem cells are rapidly lost from the niche and differentiate. The fact that somatic knock down of MAPK reduced CySC numbers by ~45% (Fig 3H), whereas all MAPK pathway mutant clones were lost, strongly suggests that CySCs lacking MAPK activity are primarily lost as a result of competition by their wild type CySC neighbors. Therefore, we conclude that MAPK signaling regulates the ability of CySCs to compete for space at the niche.

**Table 1:** Negatively marked *Egfr* or *Ras85D* mutant CySC clones are not recovered.

	2 dpci		7 dpci	
% testes with marked clone	GSCs (n)	CySCs (n)	GSCs (n)	CySCs (n)
*FRT*^*42D*^ control	68 (72)	86 (72)	63 (70)	47 (70)
*Egfr*^*124A*^	84 (64)	45 (64)	84 (49)	2 (49)
*Egfr*^*IK35*^	82 (51)	18 (51)	75 (53)	0 (53)
*FRT*^*82B*^ control	53 (19)	63 (19)	58 (36)	44 (36)
*Ras85D*^Δ*C40B*^	61 (23)	39 (23)	54 (24)	0 (24)

n: number of testes scored

Clones were negatively-marked and were identified by the absence of GFP.

### *Socs36E* mutant clones lose their competitive advantage when MAPK signaling is impaired

**Fig 5:**
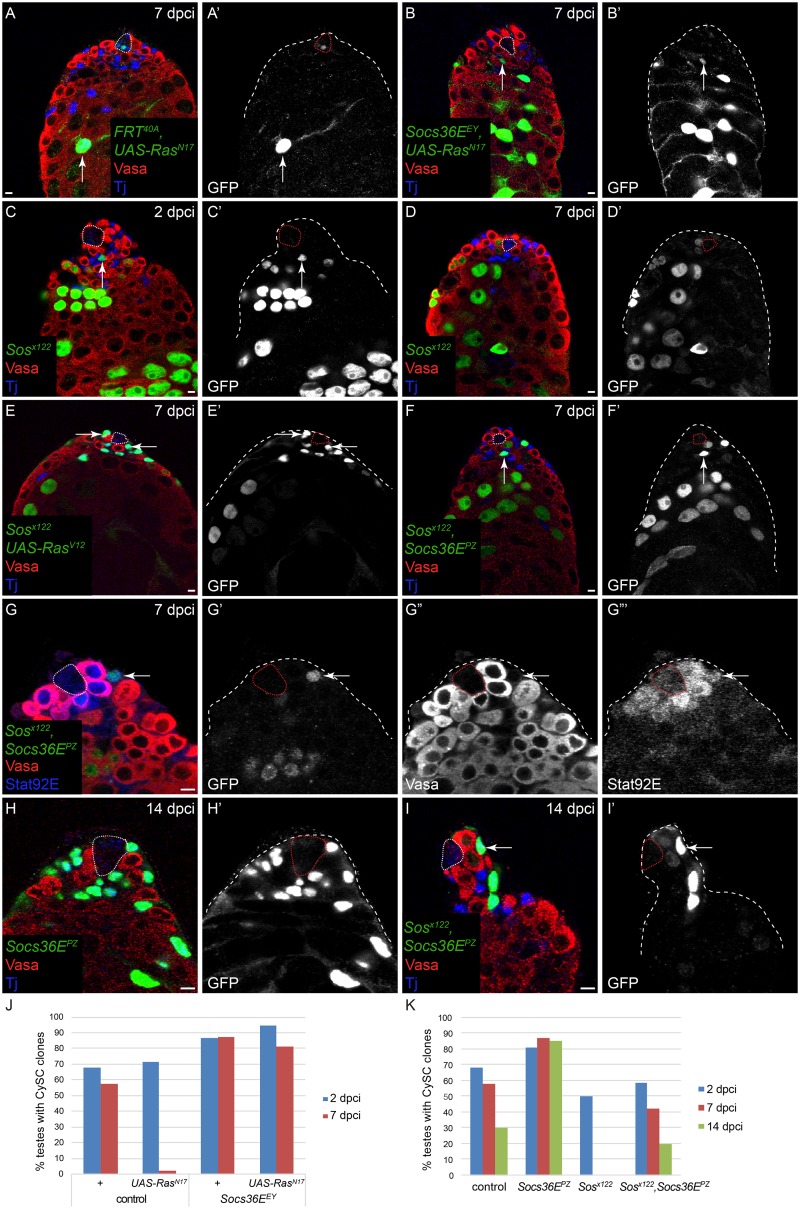
Socs36E regulates CySC competition via MAPK. All clones were generated by the MARCM technique and are positively-marked by the expression of GFP. A) *UAS-Ras*^*N17*^ CySC clones are not recovered at 7 dpci but their differentiating offspring are observed (arrow). B) By contrast, *Socs36E*, *UAS-Ras*^*N17*^ CySCs are readily recovered at 7 dpci (arrow). C, D) A *Sos* mutant CySC is recovered at 2 dpci (C, arrow) but not 7 dpci (D). E) *Sos* mutant CySCs are rescued when an activated form of Ras (Ras^V12^), which functions downstream of Sos, is also expressed in the clones (E,E’, arrows). F) *Sos*, *Socs36E* double mutant CySCs are also recovered at 7 dpci (arrow). G) *Sos*, *Socs36E* double mutant clones have robust staining for Stat92E (arrow). H) *Socs36E* mutant clones have colonized the niche at 14 dpci. I) *Sos*, *Socs36E* double mutant CySC clones are recovered at 14 dpci (arrow), indicating clone persistence. However, unlike *Socs36E* single mutant clones, *Sos*, *Socs36E* double mutant clones do not colonize the niche, indicating that MAPK signaling regulates the competitiveness of *Socs36E* mutant clones. J,K) Graphs showing CySC clone recovery rates at 2 (blue bars), 7 (red bars) and 14 (green bars in K) dpci. Whereas CySCs expressing a dominant-negative Ras (*UAS-Ras*^*N17*^) are not recovered at 7 dpci, *Socs36E*, *UAS-Ras*^*N17*^ CySCs are robustly recovered (J). In K, control CySC clones have reduced recovery rates over time, consistent with stochastic loss and replacement [18]. By contrast, *Socs36E* mutant CySCs are not lost over time, indicating their competitive advantage. While *Sos* single mutant clones are not recovered at 7 and 14 dpci, *Sos*, *Socs36E* double mutant clones are readily recovered and in a similar fashion to control clones. Vasa is red in all panels. Tj is blue in A-F, H,I. Stat92E is blue in G. The hub is outlined by a dotted line. Scale bar = 5 μM.

We have shown that Socs36E represses MAPK activity in addition to its known role in repressing JAK/STAT signaling. Therefore, we sought to clarify the relationship between Socs36E and these two signaling pathways in CySC self-renewal, in particular to establish which pathway was the functionally relevant target of Socs36E regulation. We used the MARCM technique to generate GFP-expressing clones lacking both *Socs36E* and MAPK activity and monitored the ability of these clones to self-renew and compete with wild type CySCs for niche occupancy. CySCs mutant for either of two alleles of *Socs36E* (*Socs36E*^*EY*^ and *Socs36E*^*PZ*^) self-renewed better than control clones at all time points, indicating that they are less likely to be lost through neutral competition (Fig 5J and 5K, Table 2 and [18]). We generated control clones expressing a dominant-negative form of Ras (Ras^N17^) and determined that, while they were recovered at 2 dpci, few marked CySCs were found at 7 dpci (Fig 5A and 5J, Table 2, Fisher’s exact test P<0.0001, *FRT*^*40A*^, *UAS-Ras*^*N17*^ compared to control *FRT*^*40A*^), consistent with our earlier observations that MAPK-deficient clones cannot be maintained in the niche. Surprisingly, *Socs36E* mutant clones expressing Ras^N17^ were able to self-renew and were recovered robustly at 7 dpci (Fig 5B, arrows, Fig 5J, Table 2, Fisher’s exact test P<0.0001, *Socs36E*^*EY*^ *FRT*^*40A*^, *UAS-Ras*^*N17*^ compared to *FRT*^*40A*^, *UAS-Ras*^*N17*^). One caveat of this experiment could be that MAPK activity was incompletely blocked by expression of Ras^N17^. Therefore, we generated clones that were doubly mutant for *Socs36E* and a component of the MAPK pathway. *Sos* encodes a Ras GEF and is located on the same chromosome arm as *Socs36E* (2L), enabling the generation of clones doubly mutant for *Socs36E* and *Sos* (i.e., *Socs36E* mutant cells that are deficient for MAPK signal transduction).

**Table 2:** Clone recovery rates for epistasis experiments between *Socs36E* and the JAK/STAT and MAPK pathways.

	2 dpci		7 dpci		14 dpci	
% testes with marked clone	GSCs (n)	CySCs (n)	GSCs (n)	CySCs (n)	GSCs (n)	CySCs (n)
*FRT*^*40A*^ control	56 (50)	68 (50)	58 (26)	58 (26)	55 (53)	30 (53)
*Sos*^*e26D*^	25 (36)	39 (36)	38 (53)	0 (53)	ND	ND
*Sos*^*x122*^	86 (50)	50 (50)	89 (55)	0 (55)	ND	ND
*Sos*^*x122*^, *UAS-Ras*^*V12*^	80 (10)	50 (10)	86 (37)	35 (37)	ND	ND
*Sos*^*x122*^, *UAS-P35*	ND	ND	94 (47)	0 (47)	ND	ND
*Socs36E*^*PZ*^	48 (42)	81 (42)	10 (30)	87 (30)	42 (59)	84 (59)
*Sos*^*x122*^, *Socs36E*^*PZ*^	86 (43)	58 (43)	84 (55)	42 (55)	89 (46)	20 (46)
*Socs36E*^*EY*^	29 (52)	86 (52)	31 (16)	87 (16)	38 (58)	93 (58)
*FRT*^*40A*^, *UAS-Dome*^Δ*cyt*^	40 (15)	80 (15)	59 (51)	14 (51)	ND	ND
*Socs36E*^*EY*^, *UAS-Dome*^Δ*cyt*^	14 (43)	86 (43)	29 (58)	60 (58)	ND	ND
*FRT*^*40A*^, *UAS-Stat92E RNAi*	15 (48)	83 (48)	5 (82)	0 (82)	ND	ND
*Socs36E*^*EY*^, *UAS-Stat92E RNAi*	10 (21)	95 (21)	5 (63)	19 (63)	ND	ND
*FRT*^*40A*^, *UAS-Ras*^*N17*^	57 (35)	71 (35)	75 (52)	2 (52)	ND	ND
*Socs36E*^*EY*^, *UAS-Ras*^*N17*^	19 (36)	94 (36)	25 (64)	81 (64)	ND	ND

ND: not determined. n: number of testes examined.

Clones were positively-marked and were identified by the expression of GFP.

First, we confirmed that *Sos* mutant clones were unable to self-renew using two independent alleles (Fig 5C, 5D and 5K, Table 2). In both cases mutant clones were induced and observed at 2 dpci (Fig 5C, arrow), but no mutant CySCs were recovered at 7 dpci (Fig 5D), suggesting that like other components of the MAPK pathway, Sos is required for CySC self-renewal. To verify that the lack of self-renewal observed in *Sos* mutant clones was due to loss of MAPK activity in these cells, we generated *Sos* mutant MARCM clones in which we over-expressed Ras^V12^, a dominant-active form that acts downstream of Sos. CySCs of this genotype were recovered at 60% of control rates, compared to 0% for *Sos* alone (Fig 5E, arrows, Table 2, Fisher’s exact test P<0.0001, *sos*^*x122*^ *FRT*^*40A*^, *UAS-Ras*^*V12*^ compared to *sos*^*x122*^ *FRT*^*40A*^), indicating that increasing MAPK pathway activity downstream of *Sos* is sufficient to rescue self-renewal in CySCs. We took advantage of the possibility of rescuing *Sos* mutant CySCs to determine whether cell death played a role in eliminating clones lacking MAPK activity. We expressed the baculovirus caspase inhibitor P35 to prevent apoptosis in *Sos* mutant MARCM clones and scored for CySC clones at 7 dpci. Blocking apoptosis did not increase recovery of *Sos* mutant CySCs (Table 2). Thus, caspase-dependent cell death cannot account for the loss of MAPK signaling-deficient CySCs.

Next we analyzed CySC clones that were doubly mutant for *Socs36E* and *Sos* (Fig 5F, 5I and 5K, Table 2). These mutant CySC clones were readily recovered at 7 dpci (Fig 5F, arrow, Fisher’s exact test P<0.0001, *Sos*^*x122*^, *Socs36E*^*PZ*^ *FRT*^*40A*^ compared to *Sos*^*x122*^ *FRT*^*40A*^), like CySCs lacking *Socs36E* and over-expressing dominant-negative Ras^N17^. These clones persisted for at least 2 weeks (Fig 5I and 5K), indicating long-term stem cell function. However, we noted an important difference between *Socs36E* single mutant and *Sos*, *Socs36E* double mutant CySC clones. *Socs36E* single mutant clones maintained constant clone recovery rates, indicating that they have a robust ability to bias neutral replacement and colonize the niche (Fig 5K, Table 2). Indeed, by 14 dpci, most *Socs36E* clones had entirely replaced all wild type CySCs at the niche (33/42 clones were fixed, meaning that they had colonized the entire niche, Fig 5H and S3 Table). However, recovery rates of *Sos*, *Socs36E* double mutant clones decreased over time, similar to the normal turnover observed in control clones (Fig 5K, Table 2, Fisher’s exact test at 7 dpci P = 0.0026, *Sos*^*x122*^, *Socs36E*^*PZ*^ *FRT*^*40A*^ compared to *Socs36E*^*PZ*^ *FRT*^*40A*^). In contrast to *Socs36E* mutant clones, *Sos*, *Socs36E* double mutant CySCs were not able to outcompete their neighbors and few mutant CySCs were present at the niche at 14 dpci. In these testes, wild type CySCs outnumbered mutant CySCs, and no *Sos*, *Socs36E* clones were fixed, indicating that they had not colonized the niche (Fig 5I, S3 Table, Fisher’s exact test for fixed clones P<0.0001, *Sos*^*x122*^, *Socs36E*^*PZ*^ *FRT*^*40A*^ compared to *Socs36E*^*PZ*^ *FRT*^*40A*^). Finally, we note that as in the case of Hh- and Yki-induced competition, the CySC-CySC competition caused by *Socs36E* mutation could be suppressed by removing one copy of *string (stg)*, which encodes the *Drosophila* Cdc25 protein and is a limiting factor for entry into mitosis [33]. Whereas 79% of *Socs36E* clones were fixed at 14 dpci, only 45% of *Socs36E* clones were fixed when *stg* was reduced (S3 Table, Fisher’s exact test for fixed clones P = 0.0183, *Socs36E*^*PZ*^ *FRT*^*40A*^ compared to *Socs36E*^*PZ*^ *FRT*^*40A*^; *stg/+*).

Consistent with their increased competitiveness towards CySCs, *Socs36E* single mutant clones also out-competed resident GSCs for niche space, significantly reducing GSC numbers (S3 Table, 8.4 GSCs/testis with *Socs36E* mutant clones versus 13.9 GSCs/testis with control clones at 14 dpci, P<5.2x10^-8^). As in the case of Hh- and Yki-induced CySC-GSC competition, the GSC reduction caused by *Socs36E* mutant CySCs could be suppressed by removing one copy of *string (stg)* (S3 Table, 12 GSCs/testis for *Socs36E* clones in a *stg*/+ background vs. 8.4 GSCs/testis for *Socs36E* clones in a background that was wild type for *stg*, P<0.00032). In contrast to *Socs36E* single mutant clones, the *Sos*, *Socs36E* double mutant clones did not out-compete GSCs (S3 Table, P<0.17). Notably *Sos*, *Socs36E* double mutant CySCs displayed elevated levels of stabilized Stat92E protein (Fig 5G, arrow), indicating that the JAK/STAT pathway was activated in these cells. This latter observation suggests that elevating JAK/STAT signaling is not sufficient to confer competitive ability on CySCs, consistent with our prior clonal results [18].

### *Socs36E* mutant CySCs are able to self-renew when JAK/STAT signaling is impaired

Next, we examined whether JAK/STAT pathway activity was required for self-renewal and/or competitiveness downstream of *Socs36E*. Unfortunately, there is no known JAK/STAT pathway component encoded by a gene on chromosome 2L, precluding double mutant analysis. However, we used the JAK/STAT target and effector *chinmo*, located on 2L, as a proxy for JAK/STAT activity in CySCs [34]. As previously described, *chinmo* mutant CySC clones were unable to self-renew and were likely out-competed by wild type neighbors (S2B and S2D Fig and [34,35]). Importantly, *chinmo Socs36E* double mutant clones were recovered frequently (S2C and S2D Fig), indicating that removing *Socs36E* from *chinmo* mutant CySCs restored their ability to compete with neighbors. Additionally, these double mutant clones over-proliferated and formed ectopic masses of somatic cells (S2C Fig, arrow), suggesting they were mis-specified, consistent with work showing that *chinmo* is required to maintain the male identity of CySCs [35].

**Fig 6:**
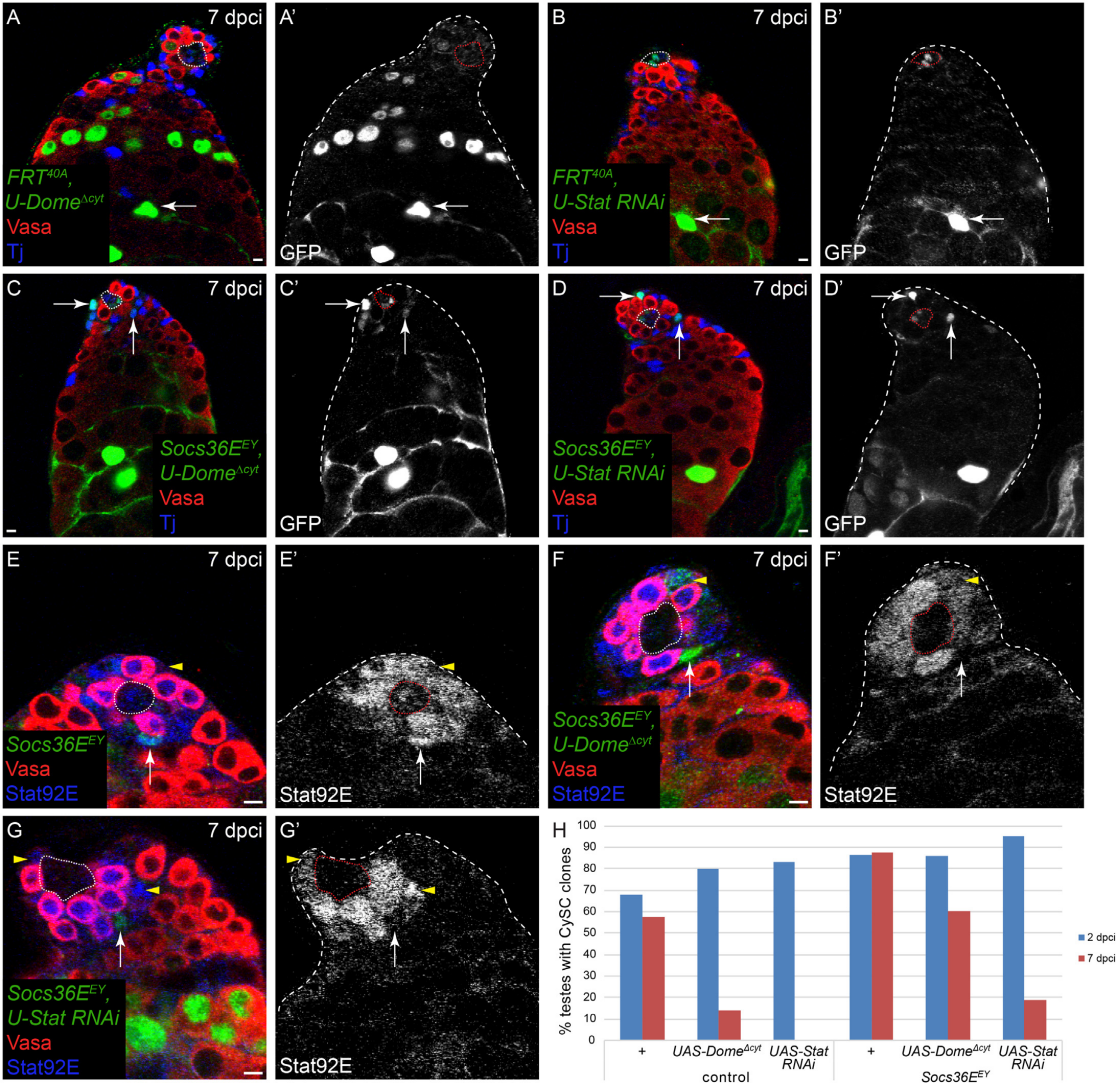
*Socs36E* mutant CySCs can self-renew without JAK/STAT signaling. All clones were generated by the MARCM technique and are positively-marked by the expression of GFP. A,B) CySC clones that express a dominant-negative form of Dome (*Dome*^Δ*cyt*^) (A), which inhibits JAK/STAT signaling, or that are depleted for Stat92E (*UAS-Stat RNAi*) (B), are not recovered at 7 dpci. However, their differentiating cyst descendants are observed at this time point (A’,B’, arrow). C,D) By contrast, when *Socs36E* is also removed from *Dome*^Δ*cyt*^ (C, arrows) or from *UAS-StatRNAi* (D, arrows) CySC clones, these clones are now maintained as stem cells. E) *Socs36E* clones have elevated levels of Stat92E (E,E’, arrow) compared with wild type CySCs (E,E’, arrowhead). Note that GSCs have higher levels of stabilized Stat92E protein than wild type CySCs. F) Some *Socs36E*, *Dome*^Δ*cyt*^ CySCs have residual levels of stabilized Stat92E (F,F’, arrowhead) and some have undetectable levels of stabilized Stat92E (F,F’, arrow). G) *Socs36E*, *UAS-Stat RNAi* CySCs have undetectable Stat92E levels (G’, arrow) whereas wild type CySCs in the same testis have moderate to high levels of Stat92E (G’, arrowheads). H) Graph of CySC clone recovery rates at 2 (blue bars) and 7 (red bars) dpci. CySCs expressing *Dome*^Δ*cyt*^ or *UAS-Stat RNAi* are not recovered at 7 dpci. By contrast, *Socs36E*, *UAS*-*Dome*^Δ*cyt*^ CySCs are recovered at robust levels at 7 dpci. *Socs36E*, *UAS-StatRNAi* CySCs are maintained at moderate levels at 7 dpci. Vasa is red in A-G. Tj is blue in A-D and Stat92E is blue in E-G. The hub is outlined by a dotted line. Scale bar = 5 μM.

In order to assess directly the role of JAK/STAT signaling downstream of *Socs36E*, we used the MARCM technique to inhibit pathway activity in *Socs36E* mutant clones. We used two approaches: first we expressed a dominant-negative form of the receptor Domeless (Dome [36]), called Dome^Δcyt^, and second we expressed an RNAi transgene against the transcription factor Stat92E. In control clones, knocking down JAK/STAT activity with either approach led to a marked loss of self-renewal: by 7 dpci very few clones expressing Dome^Δcyt^ were maintained and clonal depletion of Stat92E was sufficient to abolish self-renewal (Fig 6A, 6B and 6H, Table 2). *Socs36E* mutant CySCs that expressed Dome^Δcyt^ were recovered with high frequency, similar to controls (Fig 6C, arrows, Fig 6H, Table 2, Fisher’s exact test P<0.0001, *FRT*^*40A*^, *UAS-Dome*^Δ*cyt*^ compared to *Socs36E*^*EY*^ *FRT*^*40A*^, *UAS-Dome*^Δ*cyt*^). *Socs36E* mutant CySCs depleted for Stat92E were recovered at 7 dpci (Fig 6D, arrows, Fig 6H, Table 2, Fisher’s exact test P<0.0001, *FRT*^*40A*^, *UAS-Stat92E RNAi* compared to *Socs36E*^*EY*^ *FRT*^*40A*^, *UAS-Stat92E RNAi*), albeit at rates that were lower than control clones. Since control clones lacking Stat92E were never covered at 7 dpci, it is notable that removing *Socs36E* from these cells resulted in a moderate but significant rescue of self-renewal. The more robust rescue of self-renewal of *Socs36E*, *UAS-Dome*^Δ*cyt*^ CySCs could be due to incomplete pathway inhibition. To address this possibility, we tested whether Stat92E activity was indeed lacking in these clones by staining testes carrying *Socs36E*, *UAS-Dome*^Δ*cyt*^ or *Socs36E*, *UAS-Stat92E RNAi* clones with an antibody against stabilized, activated Stat92E [34]. In *Socs36E* mutant clones alone, as expected, we observed increased Stat92E protein (Fig 6E’, arrow, compare with wild type CySC, arrowhead). In most *Socs36E* mutant CySCs expressing Dome^Δcyt^, we observed a lack of Stat92E staining (Fig 6F’, arrow). However, a few of these CySCs displayed reduced but detectable Stat92E immunoreactivity (Fig 6F’, arrowhead), suggesting that there may be residual JAK/STAT signaling in these clones. However, in *Socs36E* mutant CySCs expressing Stat92E RNAi, we never observed any Stat92E protein (Fig 6G’ arrow, compare with wild type CySCs, arrowheads), indicating robust inhibition of JAK/STAT signaling in these clones. Although we cannot exclude the possibility that there may be some remaining Stat92E protein below the threshold of detection, these results suggest that *Socs36E* mutant CySCs are capable of renewing in the absence of Stat92E, although at reduced rates.

## Discussion

The data presented here implicate MAPK signaling as a major regulator of CySC competition for niche access and establish that the competitiveness of CySCs lacking *Socs36E* is derived primarily from their increased MAPK activity. The ability of a stem cell to self-renew reflects not only intrinsic properties but also extrinsic relationships with its neighbors [37]. For instance, if a cell is unable to compete for space at the niche then it will be no longer able to receive short-range niche signals and will be more likely to differentiate. Conversely, if a cell is more competitive for niche space, this cell and its offspring will replace wild type neighbors and colonize the entire niche [18,38,39].

Our data show that CySCs with increased MAPK signaling out-compete neighboring stem cells in CySC-CySC as well as CySC-GSC competition and that CySCs with reduced MAPK activity are themselves out-competed. We favor the interpretation that MAPK regulates primarily competitiveness rather than self-renewal because while MAPK mutant clones are lost from the niche, lineage-wide inhibition of the pathway does not result in a complete loss of stem cells. This contrasts with the role of JAK/STAT signaling in CySCs. *Stat92E* mutant CySCs are lost and lineage-wide pathway inhibition results in pronounced and rapid stem cell loss [8,12,13,40]. Based on these results, we argue that JAK/STAT signaling in CySCs primarily controls their intrinsic self-renewal capability while MAPK signaling regulates their competitiveness. Interestingly, there are important similarities between Hh and MAPK function in CySCs in that CySCs lacking Hh signal transduction are out-competed and those with sustained Hh activity out-compete wild type neighbors [14,15,18]. Lastly, we note that CySCs mutant for the tumor suppressor *Hippo* (*Hpo*) (which leads to sustained Yki activation) or *Abelson kinase* (*Abl*) also have increased competitiveness [18,19], suggesting the existence of multiple inputs controlling the ability of stem cells to stay in the niche at the expense of their neighbors. In the future, it would be interesting to determine if genetic hierarchies exist between competitive pathways or if they independently converge on similar targets. One outstanding question is how altering the competitiveness of CySCs affects the maintenance of the germ line. In the case of *Socs36E*, MAPK, Hh and Hpo, the competitive CySC displaces not only wild type CySCs but also wild type GSCs (this study and [16,18]). While these observations suggest that out-competition of CySCs and GSCs is linked, the result that *Abl* mutant CySCs only compete with CySCs and not with GSCs indicates that these two competitive processes are separable genetically [19].

It is well established that Egfr/MAPK signaling is required in somatic cells for their proper differentiation and for their encystment of the developing germ line [26,27,30,31]. In this study, we identity an additional function for Egfr/MAPK in the somatic stem cells, specifically that this pathway regulates competitiveness of CySCs, with each other and with GSCs. Regarding the latter, it is possible that the loss of GSCs when somatic cells have high MAPK signaling is linked to their possibly increased encystment by these cells. Indeed, recent work has shown that Egfr activity in CySCs regulates cytokinesis and maintenance stem cell fate in GSCs [41]. It is tempting to speculate that increased somatic Egfr activity leads to increased encystment of GSCs and loss of stem cell fate in GSCs.

**Fig 7:**
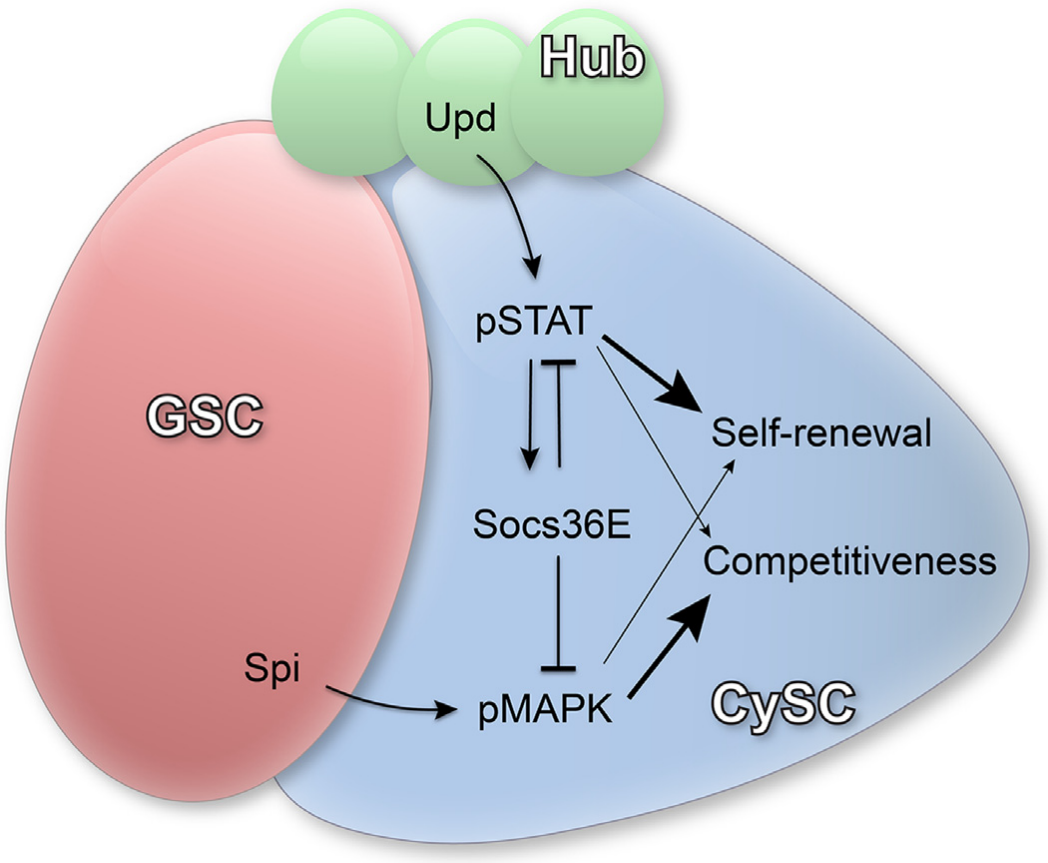
Model for the role of Socs36E in CySCs. Upd is produced by hub cells (green) and activates Stat92E (pSTAT) in CySCs (blue). JAK/STAT signaling upregulates the expression of Socs36E, which negatively regulates both JAK/STAT and MAPK signaling. pSTAT in CySCs primarily controls self-renewal (bigger arrow) but may also impact competition (smaller arrow). Spi is produced by GSCs/germ cells (red) and results in the activation of MAPK (pMAPK), which primarily regulates competitive behavior (bigger arrow) of CySCs but may also impact self-renewal (smaller arrow). In the absence of Socs36E, both JAK/STAT and MAPK pathways have elevated activity, resulting in increased self-renewal and increased competition.

MAPK may play a conserved role in niche competitiveness as mouse intestinal stem cells that acquire activating mutations in Ras bias normal stem cell replacement dynamics and colonize the niche [38,39]. Interestingly, the activating ligand Spi is produced by germ cells [26,27], suggesting that the germ line coordinates multiple behaviors in the somatic cell lineage. In addition to transducing signals from the germ line, CySCs also receive ligands from hub cells (including Hh and the JAK/STAT ligand Upd) and they have to integrate these various stimuli. If unmitigated, the combined effect of all of these signals could produce highly competitive CySCs, with overall negative effects on niche homeostasis. Our data are consistent with a model in which the induction of Socs36E by the primary self-renewal pathway (JAK/STAT) results in the restraint of a competitive trigger (MAPK) in CySCs. In this way, Socs36E acts to integrate signals from different sources and maintain homeostatic balance between resident cell populations that share a common niche (Fig 7).

## Materials and Methods

### Fly stocks and husbandry

For a full list of genotypes, see S1 Text. The following stocks are described in fFybase (flybase.org): *Egfr*^*tsla*^; *FRT*^*42D*^ *Egfr*^*IK35*^ *(Egfr*^*f2*^, Flybase); *FRT*^*82B*^ *Ras85D*^*x7b*^; *FRT*^*82B*^ *Ras85D* ^Δ*C40B*^; *Sos*^*x122*^ *FRT*^*40A*^ (gift of N. Baker); *chinmo*^*1*^ *FRT*^*40A*^; *Socs36E*^*PZ1647*^ *FRT*^*40A*^ (referred to as *Socs36E*^*PZ*^, gift of E. Matunis); *Socs36E*^*EY06665*^ *FRT*^*40A*^ (referred to as *Socs36E*^*EY*^); *Traffic jam (Tj)-Gal4*; *UAS-Rl*^*SEM*^;*UAS-λTop 4*.*4* [42]; *UAS-Rl RNAi* (MAPK RNAi, VDRC#43123); *UAS-Ras85D*^*N17*^ (gift of D. Montell); *UAS-Dome*^Δ*cyt*^ (gift of J. Hombria); *UAS-Stat92E RNAi* (BL# 33637). *FRT*^*42D*^ *Egfr*^*124A*^ and *Sos*^*e26D*^ *FRT*^*40A*^ were gifts of J. Treisman. *Egfr*^*ts*^ is *Egfr*^*tsla*^ in *trans* to *Egfr*^*124A*^. The *Sos*^*x122*^ *Socs36E*^*PZ1647*^ *FRT*^*40A*^ double mutant was generated by recombination and the presence of both mutations confirmed by the lack of complementation with *Sos*^*e26D*^ and the presence of β-gal. Crosses were maintained at 25°C except *Tj-Gal4* crosses, which were raised at room temperature and males were shifted to 29°C after eclosion for 10 days to achieve maximum Gal4 activity. For Figs 1C–1F, 4A–4D and 4F, negatively marked clones were generated by FLP/FRT [43] and clones were scored by the absence of GFP. For Figs 2D, 2E, 2G, 4E, 5 and 6, positively marked clones were generated by the MARCM technique [29] and clones were scored by the expression of GFP. All clones were induced randomly using hs-FLP by heat shocking males at 37°C for one hour. Since these techniques rely on mitotic recombination, within the somatic lineage, clones can only be induced in CySCs, which are the only mitotic somatic cells. Within the germ-line, clones can be induced in GSCs and their transit-amplifying offspring, but only GSC clones will persist. Statistical analyses were carried out using Graph Pad Prism and MS Excel.

### Immunohistochemistry

Immunohistochemistry was performed as previously described [34], except in the case of dpERK antibody, for which testes were dissected and fixed in 10 mM Tris-HCl, pH 6.8, 180 mM KCl, 50 mM NaF, 10 mM NaVO_4_ and 10 mM β-glycerophosphate as described in [26] and then treated as in the case of other antibodies. We used the following primary antibodies: guinea pig anti-Tj (1:3000, gift of D. Godt), rabbit anti-Zfh1 (1:5000, gift of R. Lehmann), rabbit anti-Stat92E (1:1000), rabbit anti-Phospho-p44/42 MAPK (Erk1/2) (Thr202/Tyr204) (1:200, Cell Signaling #9101), mouse anti-βPS Integrin, mouse anti-Eya, mouse anti-Fas3 (all 1:20, Developmental Studies Hybridoma Bank (DSHB), created by the NICHD of the NIH and maintained at The University of Iowa, Department of Biology, Iowa City, IA 52242), goat anti-Vasa (1:100, Santa Cruz Biotechnology), rabbit anti-GFP (1:500, Life Technologies), chicken anti-GFP (1:500, Aves Labs).

## Supporting Information

S1 Fig βPS-integrin is not increased at the hub-CySC interface in MAPK gain-of-function or *Socs36E* loss-of-function. A) In control testes (*Tj>+*), βPS-integrin (green) is observed in the muscle sheath as well as on somatic cell membranes. B) βPS-integrin is not increased in *Tj>λTop* testes. C,D) βPS-integrin staining is not increased in testes from *Socs36E*^*PZ*^*/+* heterozygotes (C) or in those from *Socs36E*^*PZ*^ homozygotes (D). (E) βPS-integrin staining is not increased in positively-marked *Socs36E*^*PZ*^ mutant CySC clones. In E, at 14 dpci, most of the CySCs are descendants of *Socs36E*^*PZ*^ mutant clones. βPS-integrin is green in A-D and red in E. Vasa is red and Zfh1 is blue in A-D. In E, *Socs36E* clones are green and Tj is blue. The hub is indicted by an asterisk. Scale bar = 5 μM.
(TIF)

S2 Fig Loss of *Socs36E* rescues *chinmo* mutant CySCs. A) Control CySC clones (arrow) are recovered at 7 dpci. B) By contrast, only differentiated *chinmo* mutant clones (arrow) are recovered at 7 dpci. C) *chinmo*, *Socs36E* double mutant CySC clones can be recovered at 7 dpci (C,C’, arrow), indicating that the loss of *Socs36E* rescues CySCs lacking *chinmo*. In fact, these double mutant clones aggregate and proliferate, consistent with the model that CySCs lacking *chinmo* are feminized and the lack of *Socs36E* rescues them for outcompetition. D) Graph showing CySC clone recovery rates at 2 (blue bars), 7 (red bars) and 14 (green bars) dpci for control clones, *chinmo* clones and *chinmo*, *Socs36E* double mutant clones. Removing *Socs36E* robustly rescues *chinmo* mutant CySCs at 7 dpci and these clones can still be recovered although at reduced rates at 14 dpci, indicating clone persistence. The hub is indicted by an asterisk. Scale bar = 5 μM.
(TIF)

S1 Table GSC and CySC numbers in the indicated genotypes. (DOCX)

S2 Table Positively-marked *Egfr* or *Ras85D* mutant CySC clones are not recovered. n: number of testes scored. Clones were generated by MARCM and scored by the presence of GFP. We note that the *FRT*^*82B*^ MARCM stock does not enable accurate scoring of GSC recovery rates as the UAS-GFP in this stock is not expressed well in the germ line.
(DOCX)

S3 Table Competitiveness of *Socs36E* mutant clones is suppressed by *stg* heterozygosity and *Sos* mutation. (DOCX)

S1 Text List of genotypes for each figure in the manuscript. (DOCX)

## References

[1] Losick VP, Morris LX, Fox DT, Spradling A. Drosophila stem cell niches: a decade of discovery suggests a unified view of stem cell regulation. Dev Cell. 2011;21(1):159–71. 10.1016/j.devcel.2011.06.018 21763616 PMC6894370

[2] Alexander WS, Hilton DJ. The role of suppressors of cytokine signaling (SOCS) proteins in regulation of the immune response. Annual review of immunology. 2004;22:503–29. 10.1146/annurev.immunol.22.091003.090312 15032587

[3] Shilo BZ. The regulation and functions of MAPK pathways in Drosophila. Methods. 2014;68(1):151–9. 10.1016/j.ymeth.2014.01.020 . 24530508

[4] Peterson AJ, O'Connor MB. Strategies for exploring TGF-beta signaling in Drosophila. Methods. 2014;68(1):183–93. 24680699 10.1016/j.ymeth.2014.03.016 PMC4057889

[5] Kazi JU, Kabir NN, Flores-Morales A, Ronnstrand L. SOCS proteins in regulation of receptor tyrosine kinase signaling. Cellular and molecular life sciences: CMLS. 2014;71(17):3297–310. 10.1007/s00018-014-1619-y . 24705897 PMC11113172

[6] de Cuevas M, Matunis EL. The stem cell niche: lessons from the Drosophila testis. Development (Cambridge, England). 2011;138(14):2861–9. . 21693509 10.1242/dev.056242 PMC3119301

[7] Tulina N, Matunis E. Control of stem cell self-renewal in Drosophila spermatogenesis by JAK-STAT signaling. Science (New York, NY). 2001;294(5551):2546–9. . 11752575 10.1126/science.1066700

[8] Kiger AA, Jones DL, Schulz C, Rogers MB, Fuller MT. Stem cell self-renewal specified by JAK-STAT activation in response to a support cell cue. Science (New York, NY). 2001;294(5551):2542–5. . 11752574 10.1126/science.1066707

[9] Forbes AJ, Lin H, Ingham PW, Spradling AC. hedgehog is required for the proliferation and specification of ovarian somatic cells prior to egg chamber formation in Drosophila. Development (Cambridge, England). 1996;122(4):1125–35. Epub 1996/04/01. . 8620839 10.1242/dev.122.4.1125

[10] Kawase E, Wong MD, Ding BC, Xie T. Gbb/Bmp signaling is essential for maintaining germline stem cells and for repressing bam transcription in the Drosophila testis. Development (Cambridge, England). 2004;131(6):1365–75. . 14973292 10.1242/dev.01025

[11] Shivdasani AA, Ingham PW. Regulation of stem cell maintenance and transit amplifying cell proliferation by tgf-beta signaling in Drosophila spermatogenesis. Curr Biol. 2003;13(23):2065–72. . 14653996 10.1016/j.cub.2003.10.063

[12] Leatherman JL, Dinardo S. Germline self-renewal requires cyst stem cells and stat regulates niche adhesion in Drosophila testes. Nature cell biology. 2010;12(8):806–11. 20622868 10.1038/ncb2086 PMC2917891

[13] Leatherman JL, Dinardo S. Zfh-1 controls somatic stem cell self-renewal in the Drosophila testis and nonautonomously influences germline stem cell self-renewal. Cell stem cell. 2008;3(1):44–54. 10.1016/j.stem.2008.05.001 18593558 PMC2601693

[14] Michel M, Kupinski AP, Raabe I, Bokel C. Hh signalling is essential for somatic stem cell maintenance in the Drosophila testis niche. Development (Cambridge, England). 2012;139(15):2663–9. Epub 2012/06/30. 10.1242/dev.075242 . 22745310

[15] Amoyel M, Sanny J, Burel M, Bach EA. Hedgehog is required for CySC self-renewal but does not contribute to the GSC niche in the Drosophila testis. Development (Cambridge, England). 2013;140(1):56–65. 10.1242/dev.086413 23175633 PMC3513992

[16] Issigonis M, Tulina N, de Cuevas M, Brawley C, Sandler L, Matunis E. JAK-STAT signal inhibition regulates competition in the Drosophila testis stem cell niche. Science (New York, NY). 2009;326(5949):153–6. . 19797664 10.1126/science.1176817 PMC3073347

[17] Singh SR, Zheng Z, Wang H, Oh SW, Chen X, Hou SX. Competitiveness for the niche and mutual dependence of the germline and somatic stem cells in the Drosophila testis are regulated by the JAK/STAT signaling. Journal of cellular physiology. 2010;223(2):500–10. 10.1002/jcp.22073 20143337 PMC2894562

[18] Amoyel M, Simons BD, Bach EA. Neutral competition of stem cells is skewed by proliferative changes downstream of Hh and Hpo. The EMBO journal. 2014;33(20):2295–313. 10.15252/embj.201387500 25092766 PMC4253521

[19] Stine RR, Greenspan LJ, Ramachandran KV, Matunis EL. Coordinate regulation of stem cell competition by Slit-Robo and JAK-STAT signaling in the Drosophila testis. PLoS Genet. 2014;10(11):e1004713 10.1371/journal.pgen.1004713 25375180 PMC4222695

[20] Herranz H, Hong X, Hung NT, Voorhoeve PM, Cohen SM. Oncogenic cooperation between SOCS family proteins and EGFR identified using a Drosophila epithelial transformation model. Genes & development. 2012;26(14):1602–11. Epub 2012/07/18. 10.1101/gad.192021.112 22802531 PMC3404387

[21] Almudi I, Stocker H, Hafen E, Corominas M, Serras F. SOCS36E specifically interferes with Sevenless signaling during Drosophila eye development. Developmental biology. 2009;326(1):212–23. 10.1016/j.ydbio.2008.11.014 . 19083999

[22] Callus BA, Mathey-Prevot B. SOCS36E, a novel Drosophila SOCS protein, suppresses JAK/STAT and EGF-R signalling in the imaginal wing disc. Oncogene. 2002;21(31):4812–21. 10.1038/sj.onc.1205618 . 12101419

[23] Stec W, Vidal O, Zeidler MP. Drosophila SOCS36E negatively regulates JAK/STAT pathway signaling via two separable mechanisms. Molecular biology of the cell. 2013;24(18):3000–9. 10.1091/mbc.E13-05-0275 23885117 PMC3771960

[24] Rawlings JS, Rennebeck G, Harrison SM, Xi R, Harrison DA. Two Drosophila suppressors of cytokine signaling (SOCS) differentially regulate JAK and EGFR pathway activities. BMC Cell Biol. 2004;5(1):38. 15488148 10.1186/1471-2121-5-38 PMC526380

[25] Baeg GH, Zhou R, Perrimon N. Genome-wide RNAi analysis of JAK/STAT signaling components in Drosophila. Genes & development. 2005;19(16):1861–70. 10.1101/gad.1320705 16055650 PMC1186186

[26] Schulz C, Wood CG, Jones DL, Tazuke SI, Fuller MT. Signaling from germ cells mediated by the rhomboid homolog stet organizes encapsulation by somatic support cells. Development (Cambridge, England). 2002;129(19):4523–34. . 12223409 10.1242/dev.129.19.4523

[27] Kiger AA, White-Cooper H, Fuller MT. Somatic support cells restrict germline stem cell self-renewal and promote differentiation. Nature. 2000;407(6805):750–4. . 11048722 10.1038/35037606

[28] Gabay L, Seger R, Shilo BZ. In situ activation pattern of Drosophila EGF receptor pathway during development. Science (New York, NY). 1997;277(5329):1103–6. . 9262480 10.1126/science.277.5329.1103

[29] Lee T, Luo L. Mosaic analysis with a repressible cell marker for studies of gene function in neuronal morphogenesis. Neuron. 1999;22(3):451–61. . 10197526 10.1016/s0896-6273(00)80701-1

[30] Tran J, Brenner TJ, DiNardo S. Somatic control over the germline stem cell lineage during Drosophila spermatogenesis. Nature. 2000;407(6805):754–7. . 11048723 10.1038/35037613

[31] Sarkar A, Parikh N, Hearn SA, Fuller MT, Tazuke SI, Schulz C. Antagonistic roles of Rac and Rho in organizing the germ cell microenvironment. Curr Biol. 2007;17(14):1253–8. . 17629483 10.1016/j.cub.2007.06.048

[32] Chen H, Chen X, Zheng Y. The nuclear lamina regulates germline stem cell niche organization via modulation of EGFR signaling. Cell stem cell. 2013;13(1):73–86. 10.1016/j.stem.2013.05.003 23827710 PMC3703100

[33] Neufeld TP, de la Cruz AF, Johnston LA, Edgar BA. Coordination of growth and cell division in the Drosophila wing. Cell. 1998;93(7):1183–93. . 9657151 10.1016/s0092-8674(00)81462-2

[34] Flaherty MS, Salis P, Evans CJ, Ekas LA, Marouf A, Zavadil J, et al chinmo is a functional effector of the JAK/STAT pathway that regulates eye development, tumor formation, and stem cell self-renewal in Drosophila. Dev Cell. 2010;18(4):556–68. 10.1016/j.devcel.2010.02.006 20412771 PMC2859208

[35] Ma Q, Wawersik M, Matunis EL. The Jak-STAT Target Chinmo Prevents Sex Transformation of Adult Stem Cells in the Drosophila Testis Niche. Dev Cell. 2014;31(4):474–86. 10.1016/j.devcel.2014.10.004 25453558 PMC4254588

[36] Brown S, Hu N, Hombria JC. Identification of the first invertebrate interleukin JAK/STAT receptor, the Drosophila gene domeless. Curr Biol. 2001;11(21):1700–5. . 11696329 10.1016/s0960-9822(01)00524-3

[37] Simons BD, Clevers H. Strategies for homeostatic stem cell self-renewal in adult tissues. Cell. 2011;145(6):851–62. 10.1016/j.cell.2011.05.033 21663791

[38] Vermeulen L, Morrissey E, van der Heijden M, Nicholson AM, Sottoriva A, Buczacki S, et al Defining stem cell dynamics in models of intestinal tumor initiation. Science (New York, NY). 2013;342(6161):995–8. 10.1126/science.1243148 . 24264992

[39] Snippert HJ, Schepers AG, van Es JH, Simons BD, Clevers H. Biased competition between Lgr5 intestinal stem cells driven by oncogenic mutation induces clonal expansion. EMBO reports. 2014;15(1):62–9. 10.1002/embr.201337799 24355609 PMC3983678

[40] Brawley C, Matunis E. Regeneration of male germline stem cells by spermatogonial dedifferentiation in vivo. Science (New York, NY). 2004;304(5675):1331–4. 10.1126/science.1097676 . 15143218

[41] Lenhart KF, DiNardo S. Somatic cell encystment promotes abscission in germline stem cells following a regulated block in cytokinesis. Dev Cell. 2015;34(2):192–205. 10.1016/j.devcel.2015.05.003 26143993 PMC4519359

[42] Queenan AM, Ghabrial A, Schupbach T. Ectopic activation of torpedo/Egfr, a Drosophila receptor tyrosine kinase, dorsalizes both the eggshell and the embryo. Development (Cambridge, England). 1997;124(19):3871–80. . 9367443 10.1242/dev.124.19.3871

[43] Xu T, Rubin GM. Analysis of genetic mosaics in developing and adult Drosophila tissues. Development (Cambridge, England). 1993;117(4):1223–37. . 8404527 10.1242/dev.117.4.1223

[44] Hudson AG, Parrott BB, Qian Y, Schulz C. A temporal signature of epidermal growth factor signaling regulates the differentiation of germline cells in testes of Drosophila melanogaster. PloS one. 2013;8(8):e70678 10.1371/journal.pone.0070678 23940622 PMC3734272

