# Supplementary figures and images for: Socs36E Controls Niche Competition by Repressing MAPK Signaling in the Drosophila Testis
Source: PLoS Genet. 2016 Jan 25;12(1):e1005815. doi: 10.1371/journal.pgen.1005815 (PMC4726490; doi:10.1371/journal.pgen.1005815)

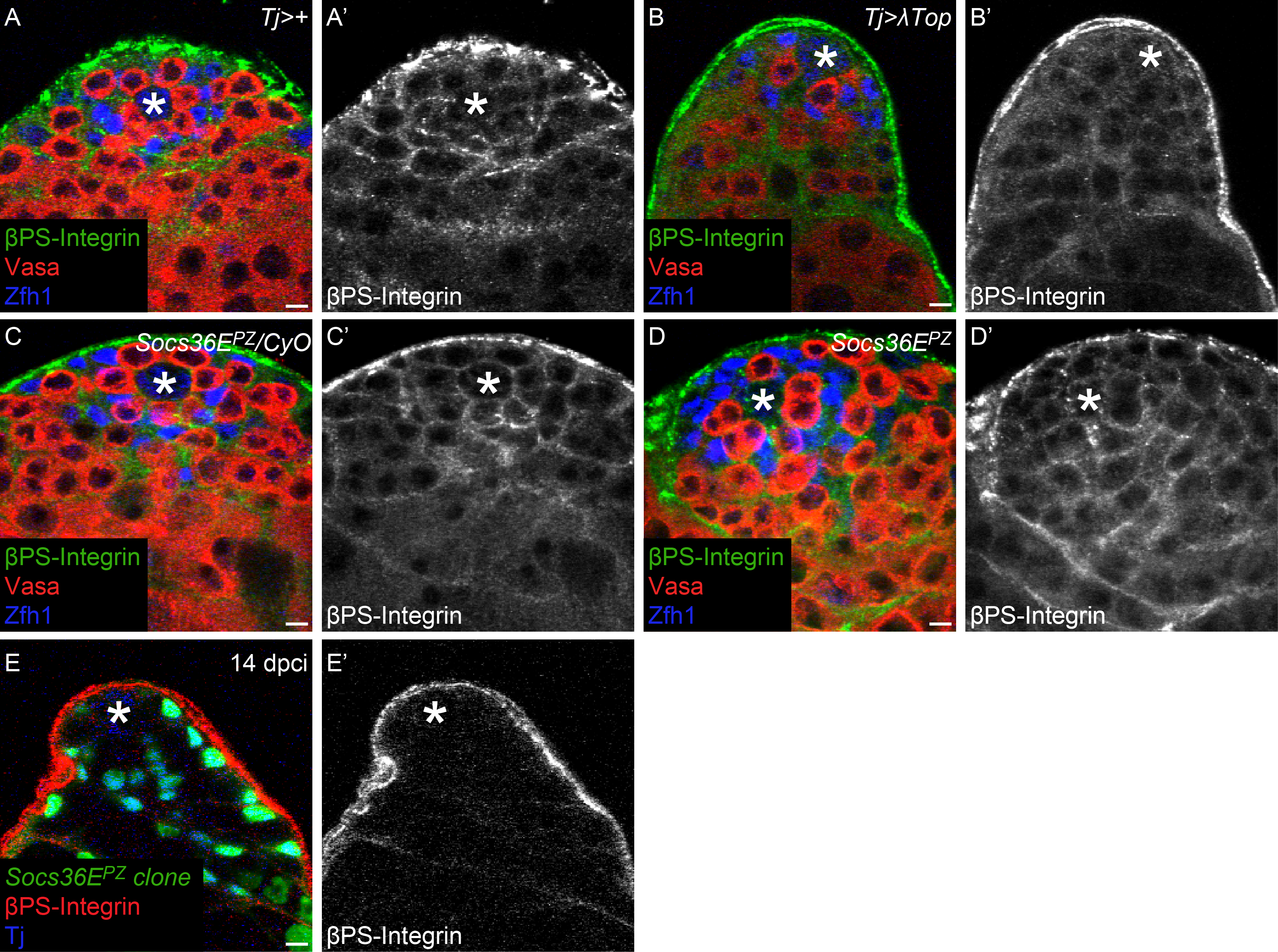

Supplement: S1 Fig — A) In control testes (Tj>+), βPS-integrin (green) is observed in the muscle sheath as well as on somatic cell membranes. B) βPS-integrin is not increased in Tj>λTop testes. C,D) βPS-integrin staining is not increased in testes from Socs36EPZ/+ heterozygotes (C) or in those from Socs36EPZ homozygotes (D). (E) βPS-integrin staining is not increased in positively-marked Socs36EPZ mutant CySC clones. In E, at 14 dpci, most of the CySCs are descendants of Socs36EPZ mutant clones. βPS-integrin is green in A-D and red in E. Vasa is red and Zfh1 is blue in A-D. In E, Socs36E clones are green and Tj is blue. The hub is indicted by an asterisk. Scale bar = 5 μM. (TIF) [file pgen.1005815.s001.tif]

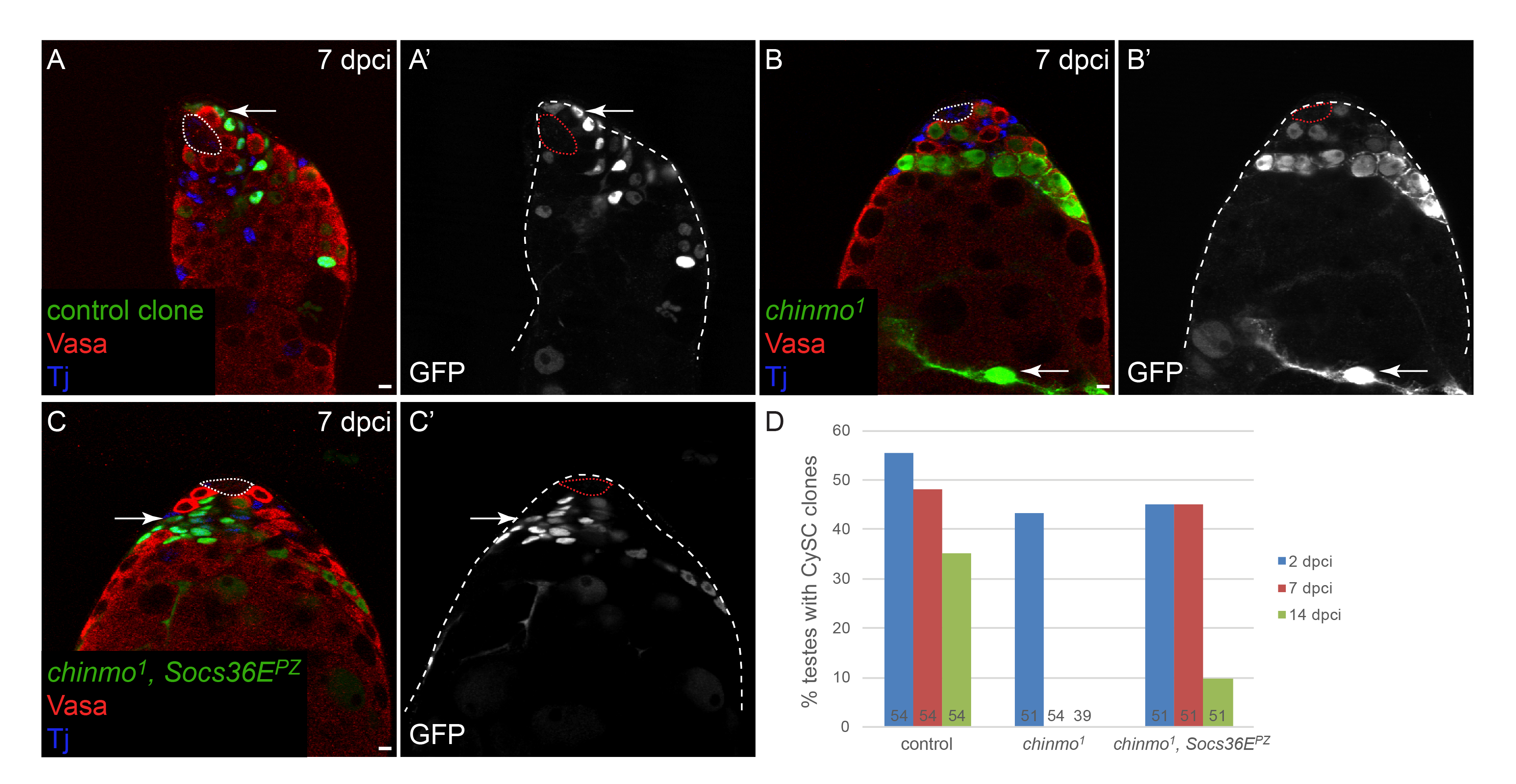

Supplement: S2 Fig — A) Control CySC clones (arrow) are recovered at 7 dpci. B) By contrast, only differentiated chinmo mutant clones (arrow) are recovered at 7 dpci. C) chinmo, Socs36E double mutant CySC clones can be recovered at 7 dpci (C,C’, arrow), indicating that the loss of Socs36E rescues CySCs lacking chinmo. In fact, these double mutant clones aggregate and proliferate, consistent with the model that CySCs lacking chinmo are feminized and the lack of Socs36E rescues them for outcompetition. D) Graph showing CySC clone recovery rates at 2 (blue bars), 7 (red bars) and 14 (green bars) dpci for control clones, chinmo clones and chinmo, Socs36E double mutant clones. Removing Socs36E robustly rescues chinmo mutant CySCs at 7 dpci and these clones can still be recovered although at reduced rates at 14 dpci, indicating clone persistence. The hub is indicted by an asterisk. Scale bar = 5 μM. (TIF) [file pgen.1005815.s002.tif]
