# Supplementary material for: Socs36E Controls Niche Competition by Repressing MAPK Signaling in the Drosophila Testis
Source: PLoS Genet. 2016 Jan 25;12(1):e1005815. doi: 10.1371/journal.pgen.1005815 (PMC4726490; doi:10.1371/journal.pgen.1005815)
Supplement: S1 Table — (DOCX) [file pgen.1005815.s003.docx]

**S1 Table : GSC and CySC numbers in the indicated genotypes.**

| Genotype | Number of GSCs  (n) | Number of CySCs  (n) |
| --- | --- | --- |
| *Socs36E^PZ^/CyO* | 13.4  (14) | 43.4  (14) |
| *Socs36E^PZ^* | 7.0  (16) | 44.75  (16) |
| *Egfr/+* | ND | 39.0  (42) |
| *Egfr^ts^* | ND | 26.8  (29) |
| *Tj>+* | 13.6  (14) | 37.8  (30) |
| *Tj>λTop* | 9.4  (21) | 59.8  (21) |
| *Tj>Rl^SEM^* | 9.8  (16) | 56.3  (16) |
| *Tj>Ras^V12^* | 1.5  (10) | ND |
| *Tj>MAPK RNAi* | ND | 21.2  (20) |
