# Supplementary material for: Socs36E Controls Niche Competition by Repressing MAPK Signaling in the Drosophila Testis
Source: PLoS Genet. 2016 Jan 25;12(1):e1005815. doi: 10.1371/journal.pgen.1005815 (PMC4726490; doi:10.1371/journal.pgen.1005815)
Supplement: S2 Table — n: number of testes scored. Clones were generated by MARCM and scored by the presence of GFP. We note that the FRT82B MARCM stock does not enable accurate scoring of GSC recovery rates as the UAS-GFP in this stock is not expressed well in the germ line. (DOCX) [file pgen.1005815.s004.docx]

**S2 Table : Positively marked *Egfr* or *Ras85D* mutant CySC clones are not recovered**.

| % testes with marked clone | 2 dpci | | 7 dpci | |
| --- | --- | --- | --- | --- |
|  | GSCs  (n) | CySCs  (n) | GSCs  (n) | CySCs  (n) |
| *FRT^42D^* control | 31  (26) | 69  (26) | 33  (40) | 60  (40) |
| *Egfr^124A^* | 55  (29) | 14  (29) | 59  (59) | 0  (59) |
| *Egfr^IK35^* | 53  (30) | 23  (30) | 58  (12) | 0  (12) |
| *FRT^82B^* control | 0  (17) | 59  (17) | 5  (39) | 51  (39) |
| *Ras85D^x7b^* | 0  (12) | 50  (12) | 7  (27) | 0  (27) |
| *Ras85D^ΔC40B^* | 0  (45) | 29  (45) | 9  (54) | 2  (54) |

n : number of testes scored. Clones were generated by MARCM and scored by the presence of GFP. We note that the *FRT^82B^* MARCM stock does not enable accurate scoring of GSC recovery rates as the UAS-GFP in this stock is not well expressed in the germ line.
