# Supplementary material for: Socs36E Controls Niche Competition by Repressing MAPK Signaling in the Drosophila Testis
Source: PLoS Genet. 2016 Jan 25;12(1):e1005815. doi: 10.1371/journal.pgen.1005815 (PMC4726490; doi:10.1371/journal.pgen.1005815)
Supplement: S3 Table — (DOCX) [file pgen.1005815.s005.docx]

**S3 Table : Competitiveness of Socs36E mutant clones is suppressed by *stg* heterozygosity and *Sos* mutation**

| Genotype | % testes with CySC clones | % CySC clones fixed | Number of GSCs when clone present | n |
| --- | --- | --- | --- | --- |
| *FRT^40A^* control | 30 | 17 | 13.9 | 53 |
| *Socs36E^PZ^* | 85 | 79 | 8.4 | 59 |
| *Socs36E^PZ^; stg^4^/+* | 91 | 45 | 12.0 | 22 |
| *Socs36E^PZ^, Sos^x122^* | 20 | 0 | 12.6 | 46 |
