# Supplementary material for: Socs36E Controls Niche Competition by Repressing MAPK Signaling in the Drosophila Testis
Source: PLoS Genet. 2016 Jan 25;12(1):e1005815. doi: 10.1371/journal.pgen.1005815 (PMC4726490; doi:10.1371/journal.pgen.1005815)
Supplement: S1 Text — (DOCX) [file pgen.1005815.s006.docx]

**Genotypes**

Figure 1.

C. *yw, hsflp^122^/Y; ubi-GFP FRT^40A^ /FRT^40A^*

D. *yw, hsflp^122^/Y; FRT^42D^ubi-GFP/FRT^42D^Egfr^IK35^*

E. *yw, hsflp^122^/Y; ubi-GFP FRT^40A^ / Socs36E^PZ1647^ FRT^40A^*

Figure 2.

A. *yw/Y; Tj-Gal4/+*

B. *yw/Y; Tj-Gal4/+; UAS-Rl^SEM^/+*

C. *yw/Y; Tj-Gal4/+; UAS-Ras85D^V12^/+*

D. *yw, hsflp^122^, UAS-nlsGFP, Tub>Gal4/Y; Tub>Gal80, FRT^40A^/FRT^40A^*

E. *yw, hsflp^122^, UAS-nlsGFP, Tub>Gal4/Y; Tub>Gal80, FRT^40A^/FRT^40A^; UAS-Ras85D^V12^/+*

F. “control” as Fig. 2A, “*UAS-Rl^SEM^*” as Fig. 2B, “*UAS-λTop*” is *yw/Y; Tj-Gal4/+; UAS-λTop/+*, “UAS-Ras^V12^” as Fig. 2C.

G. “control” as Fig. 2D

“UAS-Rl^SEM^” is *yw, hsflp^122^, UAS-nlsGFP, Tub>Gal4/Y; Tub>Gal80, FRT^40A^/FRT^40A^; UAS-Ras85D^V12^/+*

“UAS-Ras^V12^” as Fig. 2E

Figure 3.

A. w/Y; *Egfr^tsla^*/CyO or *Egfr^124A^*/CyO

B. w/Y; *Egfr^tsla^*/*Egfr^124A^*

C. as Fig. 2A

D. *yw/Y; Tj-Gal4/+; UAS-Rl RNAi/+*

E. as Fig. 2A

F. as Fig. 3D

G. “*Egfr/+*” as Fig. 3A, “*Egfr^ts^*” as Fig. 3B

H. “*Tj>+*” as Fig. 2A, “*Tj>MAPK RNAi*” as Fig. 3D, “*Tj>λTop*” as Fig. 2F, “*Tj>Rl^SEM^*” as Fig. 2B.

Figure 4.

A. *yw, hsflp^122^/Y; FRT^42D^ubi-GFP/FRT^42D^*

B. as Fig. 4A

C. as Fig. 1D

D. as Fig. 1D

E. *yw, hsflp^122^, UAS-nlsGFP, Tub>Gal4/Y; FRT^42D^ Tub>Gal80/FRT^42D^ Egfr^IK35^*

F. “*FRT^42D^* control” as Fig. 4A

“*Egfr^124A^*” is *yw, hsflp^122^/Y; FRT^42D^ubi-GFP/FRT^42D^ Egfr^124A^*

“*Egfr^IK35^*” as Fig. 1D

“*FRT^82B^* control” is *yw, hsflp^122^/Y;; FRT^82B^ubi-GFP/FRT^82B^*

“*Ras85D^ΔC40B^*” is *yw, hsflp^122^/Y;; FRT^82B^ubi-GFP/FRT^82B^ Ras85D^ΔC40B^*

Figure 5.

A. *yw, hsflp^122^, UAS-nlsGFP, Tub>Gal4/Y; Tub>Gal80, FRT^40A^/FRT^40A^; UAS-Ras85D^N17^/+*

B. *yw, hsflp^122^, UAS-nlsGFP, Tub>Gal4/Y; Tub>Gal80, FRT^40A^/ Socs36E^EY06665^ FRT^40A^; UAS-Ras85D^N17^/+*

C. *yw, hsflp^122^, UAS-nlsGFP, Tub>Gal4/Y; Tub>Gal80, FRT^40A^/Sos^x122^FRT^40A^*

D. as Fig. 5C

E. *yw, hsflp^122^, UAS-nlsGFP, Tub>Gal4/Y; Tub>Gal80, FRT^40A^/Sos^x122^FRT^40A^; UAS-Ras85D^V12^/+*

F. *yw, hsflp^122^, UAS-nlsGFP, Tub>Gal4/Y; Tub>Gal80, FRT^40A^/Sos^x122^, Socs36E^PZ1647^FRT^40A^*

G. as Fig. 5F

H. *yw, hsflp^122^, UAS-nlsGFP, Tub>Gal4/Y; Tub>Gal80, FRT^40A^/Socs36E^PZ1647^FRT^40A^*

I. as Fig. 5F

J. “control, +” as Fig. 2D

“control, *UAS-Ras^N17^*” as Fig. 5A

“*Socs36E^EY^,* +” is *yw, hsflp^122^, UAS-nlsGFP, Tub>Gal4/Y; Tub>Gal80, FRT^40A^/Socs36^EY06665^FRT^40A^*

“*Socs36E^EY^, UAS-Ras^N17^*” as Fig. 5B

K. “control” as Fig. 2D

“*Socs36E^PZ^*” as Fig. 5H

“*Sos^x122^*” as Fig. 5C

“*Sos^x122^, Socs36E^PZ^*” as Fig. 5F

Figure 6.

A. *yw, hsflp^122^, UAS-nlsGFP, Tub>Gal4/Y; Tub>Gal80, FRT^40A^/FRT^40A^; UAS-Dome^ΔCyt^/+*

B. *yw, hsflp^122^, UAS-nlsGFP, Tub>Gal4/Y; Tub>Gal80, FRT^40A^/FRT^40A^; UAS-Stat92E RNAi/+*

C. *yw, hsflp^122^, UAS-nlsGFP, Tub>Gal4/Y; Tub>Gal80, FRT^40A^/Socs36E^EY06665^FRT^40A^; UAS-Dome^ΔCyt^/+*

D. *yw, hsflp^122^, UAS-nlsGFP, Tub>Gal4/Y; Tub>Gal80, FRT^40A^/Socs36E^EY06665^FRT^40A^; UAS-Stat92E RNAi/+*

E. as Fig. 5J “*Socs36E^EY^,* +”

F. as Fig. 6C

G. as Fig. 6D

H. “control, +” as Fig. 2D, “control, *UAS-Dome^ΔCyt^*” as Fig. 6A, “control, *UAS-Stat RNAi*” as Fig. 6B, “*Socs36E^EY^,* +” as Fig. 5J “*Socs36E^EY^,* +”, “*Socs36E^EY^,* *UAS-Dome^ΔCyt^*” as Fig. 6C, “*Socs36E^EY^,* UAS-Stat RNAi” as Fig. 6D

S1 Figure.

A. as Fig. 2A

B. as Fig, 2F “*UAS-λTop*”

C. *Socs36E^PZ1647^FRT^40A^/CyO*

D. *Socs36E^PZ1647^FRT^40A^/ Socs36E^PZ1647^FRT^40A^*

E. as Fig. 5H

S2 Figure.

A. as Fig. 2D

B. *yw, hsflp^122^, UAS-nlsGFP, Tub>Gal4/Y; Tub>Gal80, FRT^40A^/chinmo^1^, UAS-CD8-GFP, FRT^40A^*

C. *yw, hsflp^122^, UAS-nlsGFP, Tub>Gal4/Y; Tub>Gal80, FRT^40A^/chinmo^1^, Socs36E^PZ1647^FRT^40A^*

D. “control” as Fig. 2D, “*chinmo^1^*” as Fig. S1B, “*chinmo^1^, Socs36E^PZ^*” as Fig. S1C

Table 1.

*FRT^42D^* control as Fig. 4A

*Egfr^124A^* as Fig. 4F

*Egfr^IK35^* as Fig. 1D

*FRT^82B^* control as Fig. 4F

*Ras85D^ΔC40B^* as Fig. 4F

Table 2:

*FRT^40A^* control as Fig. 2D

*Sos^e26D^* is *yw, hsflp^122^, UAS-nlsGFP, Tub>Gal4/Y; Tub>Gal80, FRT^40A^/Sos^e26D^FRT^40A^*

*Sos^x122^* as Fig. 5C

*Sos^x122^, UAS-Ras^V12^* as Fig. 5E

*Sos^x122^, UAS-P35* is *yw, hsflp^122^, UAS-nlsGFP, Tub>Gal4/Y; Tub>Gal80, FRT^40A^/Sos^x122^FRT^40A^; UAS-P35/+*

*Socs36E^PZ^*  as Fig. 5H

*Socs36E^PZ^, Sos^x122^* as Fig. 5F

*Socs36E^EY^* as Fig. 5J

*FRT^40A^, UAS-Dome^Δcyt^* as Fig. 6A

*Socs36E^EY^, UAS-Dome^Δcyt^* as Fig. 6C

*FRT^40A^, UAS-Stat92E RNAi* as Fig. 6B

*Socs36E^EY^, UAS-Stat92E RNAi* as Fig. 6D

*FRT^40A^, UAS-Ras^N17^* as Fig. 5A

*Socs36E^EY^, UAS-Ras^N17^* as Fig. 5B

S1 Table:

*Socs36E^PZ^*/*CyO* as Fig. S1C

Socs36E^PZ^ as Fig. S1D

*Egfr/+* as Fig. 3A

*Egfr^ts^* as Fig. 3B

*Tj>+* as Fig. 2A

*Tj>λTop* as Fig. 2F

*Tj>Rl^SEM^* as Fig. 2B

*Tj>Ras^V12^* as Fig. 2C

*Tj>MAPK RNAi* as Fig. 3D

S2 Table:

*FRT^42D^* control is *yw, hsflp^122^, UAS-nlsGFP, Tub>Gal4/Y; FRT^42D^ Tub>Gal80/FRT^42D^*

*Egfr^124A^* is *yw, hsflp^122^, UAS-nlsGFP, Tub>Gal4/Y; FRT^42D^ Tub>Gal80/FRT^42D^ Egfr^124A^*

*Egfr^IK35^* as Fig. 4E

*FRT^82B^* control is *yw, hsflp^122^, UAS-nlsGFP/Y; ; Tub>Gal4 FRT^82B^ Tub>Gal80/FRT^82B^*

*Ras85D^x7b^* is *yw, hsflp^122^, UAS-nlsGFP/Y; ; Tub>Gal4 FRT^82B^ Tub>Gal80/FRT^82B^ Ras85D^x7b^*

*Ras85D^ΔC40B^* is *yw, hsflp^122^, UAS-nlsGFP/Y; ; Tub>Gal4 FRT^82B^ Tub>Gal80/FRT^82B^ Ras85D^ΔC40B^*

S3 Table:

*FRT^40A^* control as Fig. 2D

*Socs36E^PZ^* as Fig. 5H

*Socs36E^PZ^; stg^4^/+* is *yw, hsflp^122^, UAS-nlsGFP, Tub>Gal4/Y; Tub>Gal80, FRT^40A^/Socs36E^PZ1647^FRT^40A^*; *stg^4^, e/+*

*Socs36E^PZ^, Sos^x122^* as Fig. 5F
